# Causal Inference Regarding Infectious Aetiology of Chronic Conditions: A Systematic Review

**DOI:** 10.1371/journal.pone.0068861

**Published:** 2013-07-25

**Authors:** Sofia Orrskog, Emma Medin, Svetla Tsolova, Jan C. Semenza

**Affiliations:** 1 Heron Evidence Development AB, Stockholm, Sweden; 2 Karolinska Institutet, Stockholm, Sweden; 3 European Centres for Disease Prevention and Control, Stockholm, Sweden; Drexel University College of Medicine, United States of America

## Abstract

**Background:**

The global burden of disease has shifted from communicable diseases in children to chronic diseases in adults. This epidemiologic shift varies greatly by region, but in Europe, chronic conditions account for 86% of all deaths, 77% of the disease burden, and up to 80% of health care expenditures. A number of risk factors have been implicated in chronic diseases, such as exposure to infectious agents. A number of associations have been well established while others remain uncertain.

**Methods and Findings:**

We assessed the body of evidence regarding the infectious aetiology of chronic diseases in the peer-reviewed literature over the last decade. Causality was assessed with three different criteria: First, the total number of associations documented in the literature between each infectious agent and chronic condition; second, the epidemiologic study design (quality of the study); third, evidence for the number of Hill's criteria and Koch's postulates that linked the pathogen with the chronic condition.

We identified 3136 publications, of which 148 were included in the analysis. There were a total of 75 different infectious agents and 122 chronic conditions. The evidence was strong for five pathogens, based on study type, strength and number of associations; they accounted for 60% of the associations documented in the literature. They were human immunodeficiency virus, hepatitis C virus, *Helicobacter pylori*, hepatitis B virus, and *Chlamydia pneumoniae* and were collectively implicated in the aetiology of 37 different chronic conditions. Other pathogens examined were only associated with very few chronic conditions (≤3) and when applying the three different criteria of evidence the strength of the causality was weak.

**Conclusions:**

Prevention and treatment of these five pathogens lend themselves as effective public health intervention entry points. By concentrating research efforts on these promising areas, the human, economic, and societal burden arising from chronic conditions can be reduced.

## Introduction

Over the last decades, the global disease burden has continued to shift away from communicable to non-communicable diseases and from premature death to years lived with disability [1]. Currently, chronic conditions account for almost 60% of all deaths and 43% of the global burden of disease and by 2020 their contribution is expected to rise to 73% and 60%, respectively. Most importantly, 79% of deaths attributed to these conditions occur in developing countries. These challenges loom large on the horizon and call for prevention and early treatment of chronic diseases in order to attenuate the human, economic and societal costs [2]. However, tackling this predicament entails better understanding of the natural history of chronic diseases, particularly of the early preclinical phase. Infectious agents as the aetiologic origin of chronic conditions have been suspected and investigated since the last century [2]. To date, a number of associations between infectious pathogens and chronic conditions have been established unequivocally. These include the associations of *Helicobacter pylori* with peptic ulcer and gastric cancer, hepatitis B virus or hepatitis C virus (HBV or HCV, respectively) with liver cirrhosis and cancer, and human papilloma virus (HPV) with cervical cancer [3]. Conversely, data on other chronic conditions (e.g., multiple sclerosis and Crohn's disease) indicate an infectious aetiology but a definite causative infectious agent has eluded detection. For many other chronic conditions, the evidence of an infectious aetiology is weak or even conflicting, e.g., the role of *Chlamydia pneumoniae* in the aetiology of atherosclerosis [3].

The technical difficulties for establishing causal links are substantial. Infectious agents are often cleared from the host before symptoms arise, making detection difficult and traditional diagnostic methods might not be appropriate for the detection and isolation of novel or rare pathogens. A multitude of factors related to the infection may increase the risk of developing a chronic condition: Host characteristics (e.g., age, sex, genetic profile, co-morbidities, and immune status), type of agent (e.g., biologic, chemical, physical, and nutritional) and environmental factors (e.g., pollution, radiation, and food). These factors might interact in a synergistic way which multiplies disease risk (e.g., aflatoxin and HBV in the aetiology of liver cancer). In addition, co-infections or recurrent infections might also be determinants of disease. Finally, current diagnostic methods may not be appropriate for the detection and isolation of novel or rare infectious agents, although molecular methods have been improved and refined over the years and, therefore, have increased the sensitivity and specificity of such methods. Thus, building a solid evidence-base for many of these associations has proven difficult at best. However, mounting effective interventions hinges on available evidence for novel associations that can then be translated into prevention and treatment strategies [2]. Insights into the natural history of disease can help advance prevention and treatment strategies such as vaccination or antimicrobial treatment. Since chronic conditions last for extended periods of time and tend to incur increasing health-care costs related to treatment and lifelong chronic medical care, such interventions may prove to be highly cost effective.

Thus, the objective of this study was to assess the evidence for the infectious aetiology of chronic conditions in order to pinpoint the most promising prevention and treatment strategies. Confirming, refuting, or modifying the involvement of infectious agents in idiopathic chronic conditions will significantly advance prevention and/or treatment of these infections [2]. By concentrating research efforts on a few promising areas, the human, economic, and societal burden arising from chronic conditions can be reduced. In light of the shifting epidemiology of the global burden of disease, prevention of chronic conditions has become a public health priority.

In order to tackle this issue and advance the field, we systematically reviewed all infectious agents that have been implicated as aetiologic agents of chronic conditions in the peer-reviewed literature over the last decade. Data on causality was extracted from these papers to assess the strength of association based on the quantity of associations documented in the literature, the quality of the evidence (study design) and the number of causality criteria fulfilled, specifically Hill's criteria of causation and Koch's postulates.

## Methods

### Search strategy and selection criteria

A protocol was devised for the systematic study, outlining the scope, inclusion and exclusion criteria, data sources, and specifications for data extraction. The searches were performed in the MEDLINE®, Embase®, Cochrane Reviews and Cochrane Clinical Trials databases, and publications were ordered via the British Library. The full search strategy can be found in Table S1 and S2. The inclusion criteria were as follows: infectious agent implicated as a cause or a co-factor of a chronic condition (chronic conditions were defined as diseases, disabilities and sequelae that last at least three months); publication in English between January 2001 and November 2010; study types, i.e. systematic reviews (SRs) randomised controlled trials (RCTs), cohort studies, cross-sectional studies, case-control studies, case studies/case series/case reports, non-systematic literature reviews, or pathological assessments of diseased material. Exclusion criteria were: Infectious agents described as triggers to chronic conditions; non-English language; publication year before 2001; study type, i.e. other studies than those outlined in the inclusion criteria above (including animal studies).

### Systematic review methodology

Bibliographic details and abstracts of all citations detected by the literature search were downloaded into the HERON Systematic Review Database, a bespoke, SQL-based internet database. Citations were first screened based on the abstract supplied with each citation by two independent reviewers. Those that did not match the eligibility criteria were excluded at this ‘first pass’. Full-text copies of all references that could potentially meet the eligibility criteria were ordered, including studies where it was not possible to exclude citations only based on the abstract. The eligibility criteria were applied to the full-text citations (‘second pass’) by two independent reviewers, and the data presented in the publications included after this stage were extracted by two independent reviewers. For first pass, second pass, and data extraction, any discrepancies between the two reviewers were reconciled by a third reviewer. The critical appraisal of the included publications was conducted using study design-specific Scottish Intercollegiate Guidelines Network (SIGN) checklists [4]. In addition, two recent textbooks were used to validate the extracted data [3], [5]. These textbooks were chosen because they were well aligned with the scope of the current study.

### Assessment of strength of evidence

To facilitate the presentation of the data, the chronic conditions were grouped into disease areas according to the highest hierarchy level in the International System for Classification of Diseases (ICD)-10, the Major Diagnostic Categories (MDCs) [6]. In the following sections, the term ‘disease areas’ refers to the MDC groups.

Strength of associations, i.e. the degree of relationship between infectious agents and chronic conditions, was assessed using three different methods. First, the total number of associations identified between each infectious agent and chronic condition was quantified; replication of findings in different studies was considered an indication of epidemiologic consistency. One standard deviation from the mean for the number of associations in each MDC group was used to identify the cut-off level for whether the association was defined as well established or not well established.

Second, study design was taken into account. Higher ranking in the Australian National Health and Medical Research Council (NHMRC) evidence hierarchy for studies on aetiology was indicative of stronger associations [7]. Each study design was assessed according to its place in the research hierarchy. The hierarchy reflects the potential of each study to adequately answer a particular research question, while minimising potential biases. Systematic reviews and prospective cohort studies are ranked the highest according to the NHMRC evidence hierarchy for studies on aetiology, followed by retrospective cohort studies, case-control studies, and case studies/series/reports. Randomised controlled trials (RCTs) were not included in the NHMRC evidence hierarchy but were ranked as level II studies in the current study as RCTs generally belong to that evidence level. Non-systematic literature reviews were also not included in the NHMRC evidence hierarchy but were included in this paper as the lowest evidence level (level IV). Associations presented in systematic reviews, prospective cohort studies and RCTs were defined as strong (NHMRC level I or II) whereas associations described in the context of retrospective cohort studies, case-control studies, and case studies/series/reports (NHMRC level III) were defined as weak.

Third, the number of Hill's criteria of causation and Koch's postulates that were fulfilled by the study were extracted. Each study and association was individually assessed and qualitative data were extracted from each paper according to an extraction table described in the study protocol. For this assessment, higher number of conditions fulfilled indicated stronger associations. Hill's criteria of causation and Koch's postulates complement each other and are commonly used to determine strength of associations and causality [8]. Hill's criteria of causation include six questions on biological plausibility, dose-response (natural vs. interventional), strength and specificity of the association, consistency of the results across different research groups, and temporality. Hill's criteria have been developed for epidemiological studies [8], [9]. Hill's criteria regarding biological plausibility and specificity are considered to be of less importance when establishing causality [9] and, therefore, information on these criteria were not extracted. Also, Hill's criterion regarding strength of evidence was not included in the analysis as it was a more subjective measure and could not be answered with a ‘Yes’ or ‘No’. Thus, only four of Hill's criteria were extracted and included in the analysis. Koch's postulates, originally developed for experimental studies, contain four questions regarding presence of infectious agents in diseased individuals and tissues as well as inoculation and reisolation of the infectious agent in an experimental setting. All four of Koch's postulates were extracted from the studies included in the analysis. It is generally conceived that all of Koch's postulates should be fulfilled in order for an association to be considered strong [8]. However, given that primarily epidemiological studies were included in the current study, it was anticipated that only the first two of Koch's postulates (i.e., presence of infectious agents in diseased individuals and tissues) would be fulfilled. Thus, in the analysis, in order for an association to be considered strong, at least two of Koch's postulates had to be fulfilled. If less than two of Koch's postulates were fulfilled, the association was considered weak. No thresholds were applied to the four extracted Hill's criteria of causation as there are no thresholds generally accepted in the literature [8], [9].

## Results

### Systematic searches

**Figure 1:**
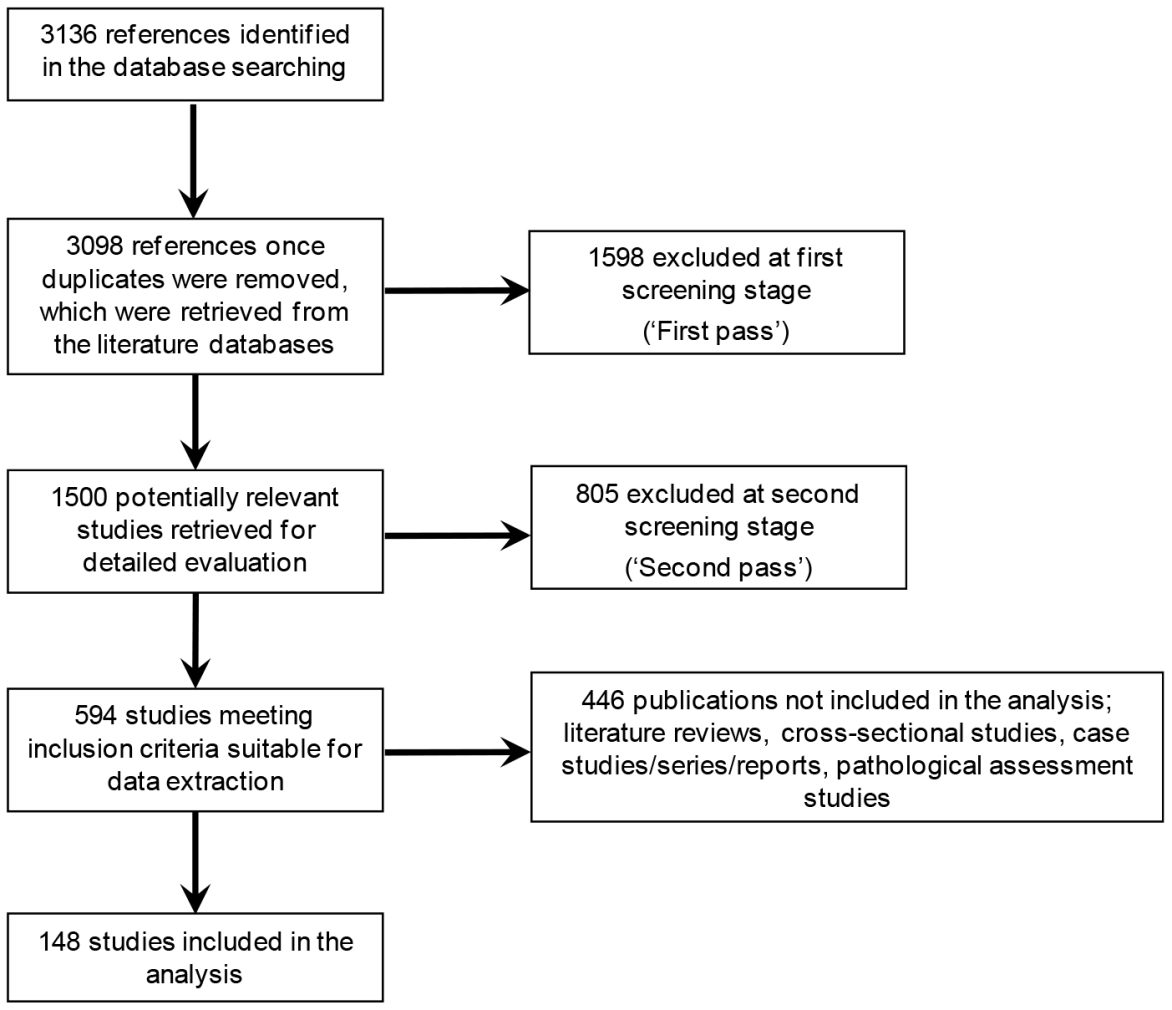
Flow diagram outlining included and excluded publications (year 2001–2010). A protocol was devised for the systematic study, and the searches were performed in the MEDLINE®, Embase®, Cochrane Reviews and Cochrane Clinical Trials databases. The following inclusion criteria were applied: Infectious agent implicated as a cause or a co-factor of a chronic condition (i.e. disease, disabilities and sequelae that last at least three months); publication in English between 2001 and 2010; study types, i.e. SRs; RCTs, cohort studies, cross-sectional studies, case-control studies, case studies/case series/case reports, literature reviews, or pathological assessments of diseased material. Exclusion criteria were: Infectious agents described as triggers to the chronic condition; non-English language; publication year before 2001; study type, i.e. other studies than those outlines in the inclusion criteria above (including animal studies). In total, 3136 publications were identified in the systematic searches in the databases, and 148 publications were finally included in the analysis. Citations were first screened by two independent reviewers (‘first pass’). Full-text copies of all references that could potentially meet the eligibility were then screened by two independent reviewers (‘second pass’). The data presented in the publications included after this stage were extracted by two independent reviewers.

The systematic searches were conducted in November 2010 and resulted in the identification of 3136 publications (Figure 1). 1500 publications were considered relevant at ‘first pass’ and 594 publications after review of the full text versions of the studies. As there are no SIGN checklists available for cross-sectional studies, case studies/case series/case reports, non-systematic literature reviews and pathological assessments of diseased material the findings from these study types were excluded from the current analysis (an overview of the associations identified in these studies can be found in Table S3). Thus, 148 publications of the study types SRs, RCTs, prospective cohort studies, retrospective cohort studies, and case-control studies were included in the analysis (Figure 1). In these publications, 408 individual associations were described in total (Table S2) [10]–[157].

The 148 publications included 20 SRs, as well as 8 RCTs, 46 prospective cohort studies, 31 retrospective cohort studies, and 43 case-control studies. The majority of methodological aspects included in the SIGN checklists were addressed in the systematic reviews, RCTs, and case-control studies, which indicated that the overall quality of these publications was good; in 75% of the systematic reviews, 61% of the RCTs, and in 59% of the case-control studies, the aspects of the SIGN checklists evaluated were ‘well covered’ or ‘adequately addressed’. However, in only 33% of the prospective and retrospective cohort studies, the aspects of the SIGN checklists evaluated were ‘well covered’ or ‘adequately addressed’, indicating that these publications were of lower quality.

### Publication frequency by disease area

**Figure 2:**
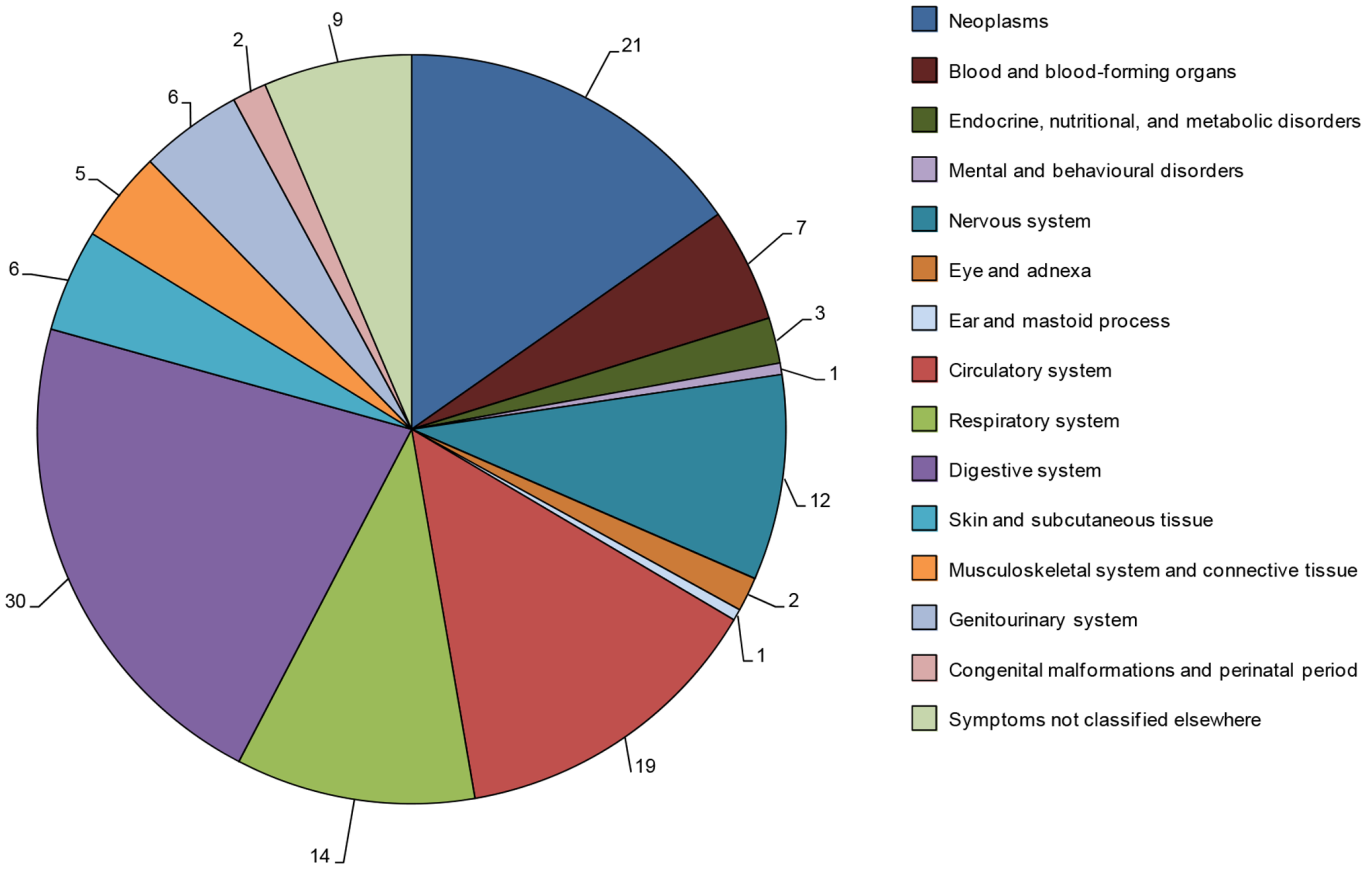
Percentage of publications per disease area (year 2001–2010). The chronic conditions were grouped into disease areas according to the highest hierarchy level in the ICD-10, and are displayed in that order. As certain publications described several individual associations, the classification into disease areas is not exclusive. Thus, the total percentage does not sum up to 100%. The disease areas where the highest numbers of publications were identified were the digestive system, neoplasms, the circulatory system, the respiratory system, and the nervous system.

The majority of associations between infectious agents and chronic conditions identified were concentrated on a few disease areas. Chronic conditions of the digestive system, circulatory system, respiratory system, nervous system, and neoplasms accounted for almost 70% of the total number of associations identified. The percentage of publications describing individual associations within these disease areas was 30% for the digestive system, 21% for neoplasms, 19% for the circulatory system, 14% for the respiratory system, and 12% for the nervous system (some publications reported associations in several disease areas) (Figure 2). There were 9% of the publications or less describing the remaining 30% of associations between infectious agents and each of the remaining disease areas, such as endocrine, nutritional, and metabolic disorders, mental and behavioural disorders, and musculoskeletal system and connective tissue.

### Ranking the weight of evidence

**Figure 3:**
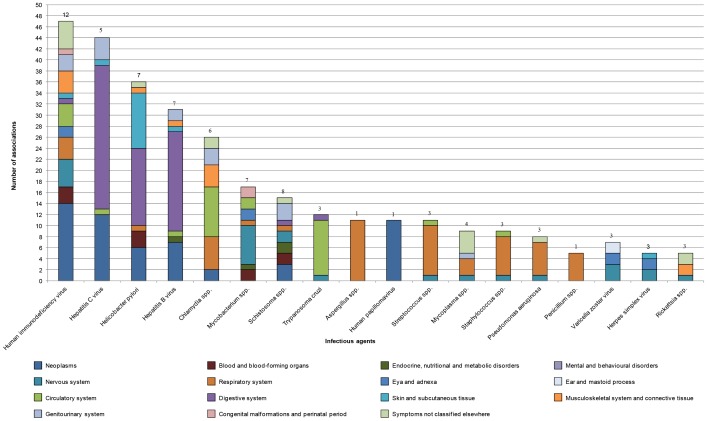
Number of associations described per infectious agent (year 2001–2010). There were 75 infectious agents identified in this study. At least five associations to one or more disease areas were identified for each of the infectious agents displayed. The number of associations per infectious agent was disaggregated into disease areas based on the highest hierarchy level in the ICD-10, and is displayed above the bars. The infectious agents for which the highest numbers of associations were described were HIV, HCV, *Helicobacter spp.*, HBV, and *C. pneumoniae*. Generally, viruses tended to be associated with a high number of disease areas, followed by bacteria, parasites, and fungi.

In total, there were 75 different infectious agents and 122 different chronic conditions identified in the systematic study. An analysis of the number of associations identified per infectious agent demonstrated that human immunodeficiency virus (HIV) was associated with many different chronic conditions, neoplasms being the largest; HCV and *Helicobacter* spp. and HBV were mainly involved in diseases of the digestive system, and *C. pneumoniae* involved in endocrine, nutritional and metabolic diseases (Figure 3). The number of individual associations described for these infectious agents ranged from 26 for *C. pneumoniae* to 47 for HIV. The evidence was strong for these five pathogens; they accounted for 60% of the associations documented in the literature. With the exception of *Mycobacteria* spp. and *Schistosoma* spp. all other pathogens examined were only associated with very few chronic conditions (≤3) and when applying the three different lines of evidence, the strength of the individual associations was weak.

The versatility of the infectious agents affecting different disease areas is illustrated in Figure 3. A breakdown by disease area reveals three broad groups of pathogens: low-specificity pathogens impacting a large number of disease areas (e.g., HIV with 12 disease areas); medium-specificity pathogens impacting around 7 disease areas (e.g., HCV, HBV, and *Helicobacter* spp.) and high-specificity pathogens affecting only one disease area (e.g., HPV, and *Aspergillus* spp.). Generally, viruses were associated with a high number of disease areas, followed by bacteria, parasites, and fungi.

**Figure 4:**
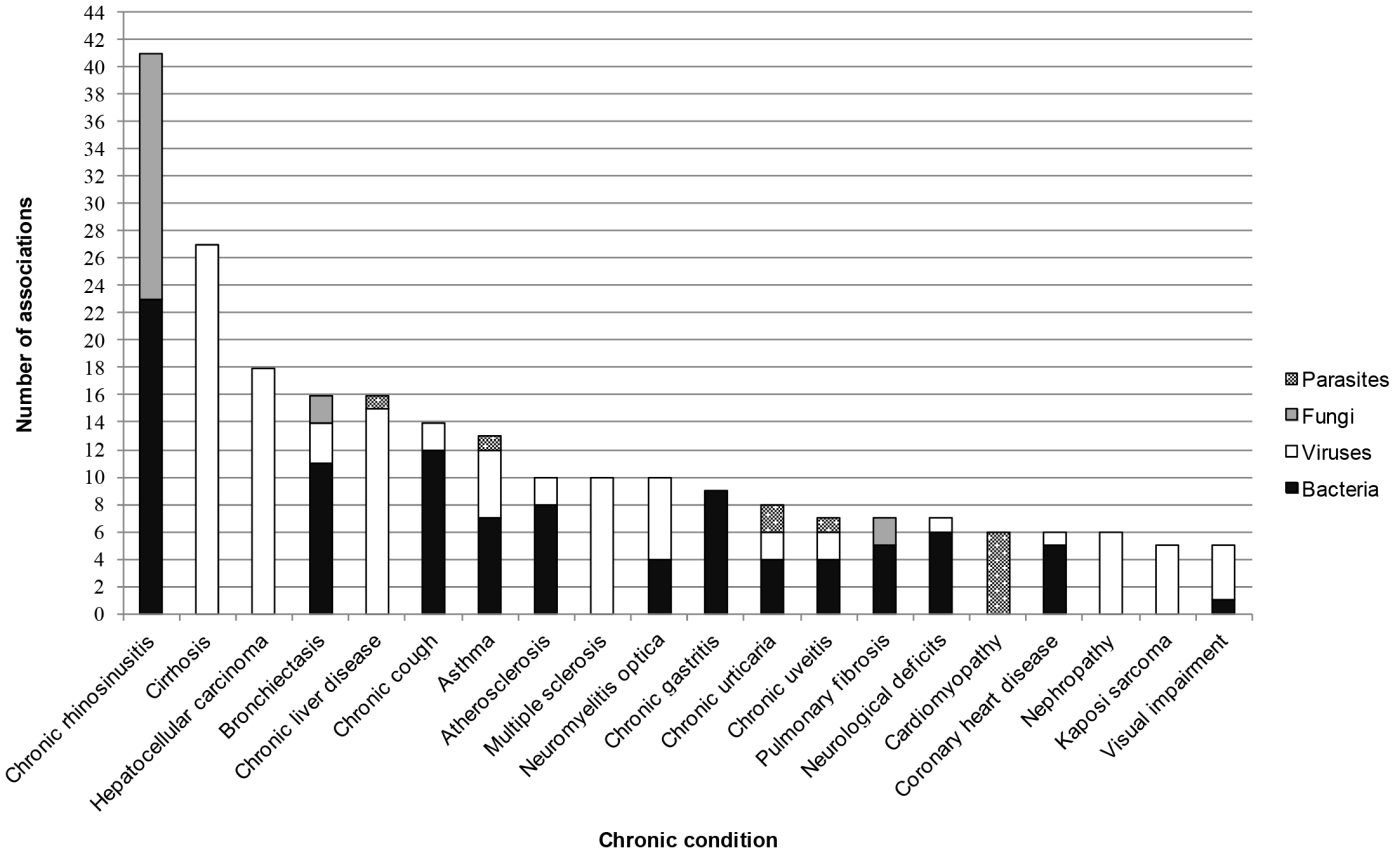
Number of associations per chronic condition (year 2001–2010). There were 122 chronic conditions identified in the current study. At least five associations were identified for each of the chronic conditions displayed, and the number of associations per chronic condition was disaggregated according to the class of infectious agent (i.e. bacteria, viruses, fungi, and parasites). The top-five chronic conditions identified in this data set were chronic rhinosinusitis, cirrhosis, hepatocellular carcinoma, bronchiectasis, and chronic liver disease. Viruses were most frequently associated with these chronic conditions, followed by bacteria, fungi, and parasites.

Infectious agents were categorised into classes (bacteria, viruses, fungi, and parasites; for a full list see Table S4) and mapped according to the number of associations per chronic conditions (Figure 4). In general, viruses were most frequently associated with these chronic conditions, followed by bacteria, fungi, and parasites, in part due the fact that viruses have been studied extensively and are relatively easily detectable. The highest number of individual associations between an infectious agent and a chronic condition was found for certain chronic conditions of the respiratory and digestive systems (Figure 4): chronic rhinosinusitis was generally associated with bacteria and fungi; cirrhosis and hepatocellular carcinoma were associated with viruses; bronchiectasis with bacteria; chronic liver disease with viruses; and chronic cough and asthma predominantly with bacteria and viruses.

### Assessment of strength of associations

**Figure 5:**
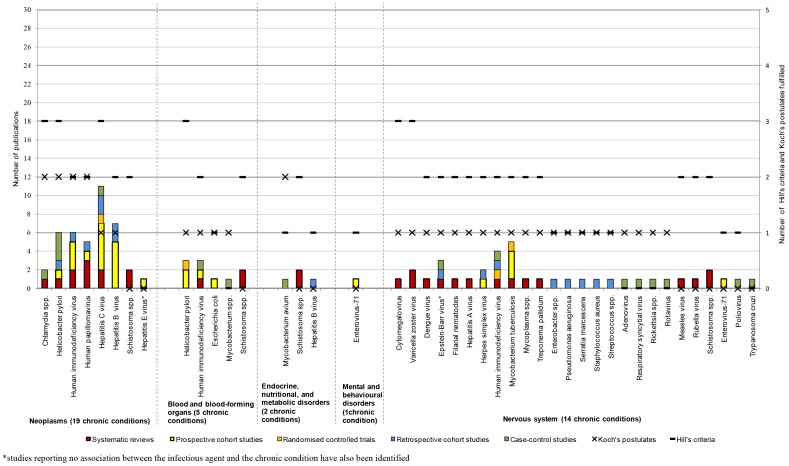
Number of publications per study type for neoplasms, blood and blood-forming organs, endocrine, nutritional and metabolic disorders, and nervious system (year 2001–2010). The chronic conditions were grouped into disease areas according to the highest hierarchy level in the International System for Classification of Diseases (ICD)-10. Associations between infectious agents and chronic conditions belonging to 15 disease areas were identified (Figures 5, 6, 7, 8). The numbers of publications per infectious agent were quantified and, for each infectious agent, the publications were presented according to study type (i.e., systematic reviews, prospective cohort studies and RCTs, retrospective cohort studies, and case-control studies). For each infectious agent and disease area, the total numbers of Hill's criteria and Koch's postulates fulfilled were determined. In the figures, the infectious agents were ranked, first according to the number of Koch's postulates fulfilled and secondly according to the number of Hill's criteria fulfilled. In this dataset, the strongest associations were those between chronic conditions of the circulatory system and *C. pneumoniae* or *T. cruzi*, and between chronic conditions of the genitourinary tract and *C. trachomatis*.

**Figure 6:**
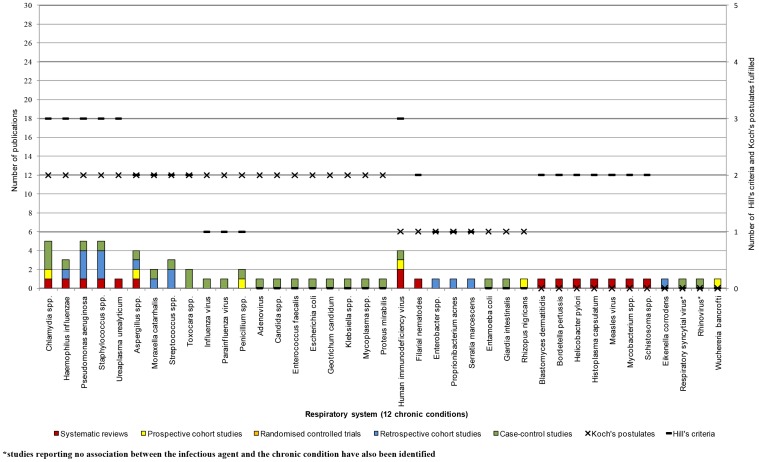
Number of publications per study type for the respiratory system (year 2001–2010).

**Figure 7:**
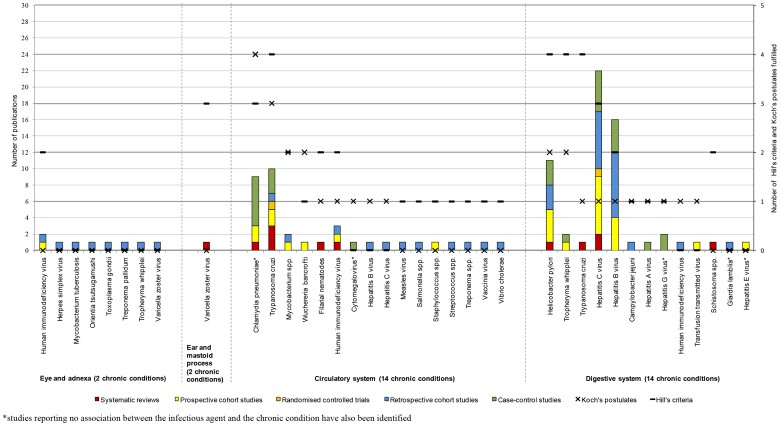
Number of publications per study type for eye and adnexa, ear and mastoid process, circulatory system, digestive system (year 2001–2010).

**Figure 8:**
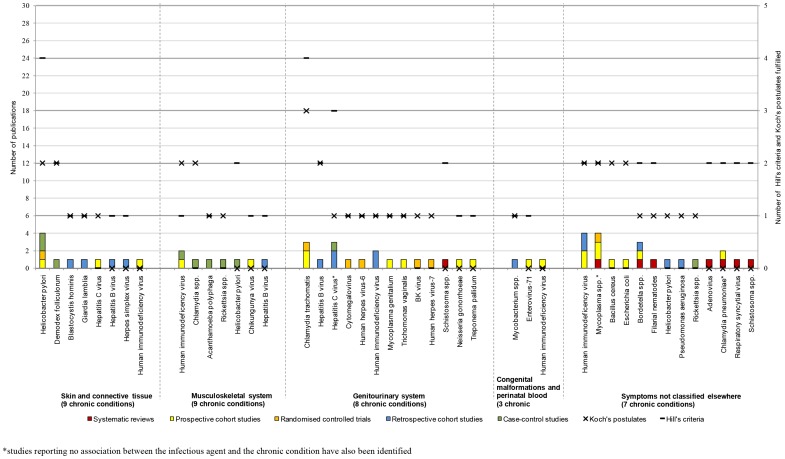
Number of publications per study type for skin and connective tissue, muscoskeletal system, genitourinary system, congenital malformations and perinatal blood, unclassified symptoms (year 2001–2010).

The analysis based on the number of studies and study type demonstrated that the strongest associations were those between HPV or HIV and neoplasms [11], [25], [51], [69], [72], [75], [77], [83], [86], [94], [96], [102], [106], [107], [114], [115], [123], [125], [151], [157], as well as between HCV or *H. pylori* and chronic conditions of the digestive system [16], [18], [21], [23], [28], [31], [47], [56], [75], [77], [80], [81], [83], [91], [96], [98]–[102], [106], [107], [109], [110], [116], [123], [130], [134], [148], [151], [152], [154], [157]. However, for the majority the disease areas, few associations to infectious agents were identified and few of Koch's postulates and Hill's criteria were fulfilled (Figures 5, 6, 7, 8). For the following disease areas, there were no or few associations where at least one of Hill's criteria and Koch's postulates were fulfilled and there were only few studies identified that described the associations: Blood and blood-forming organs, endocrine, nutritional, and metabolic diseases, mental and behavioural disorders, eye and adnexa, ear and mastoid process, musculoskeletal system and connective tissue, and congenital malformations and perinatal period.

The analysis based on the number of Hill's criteria and Koch's postulate demonstrated that the associations between chronic conditions of the circulatory system and *C. pneumoniae* or *Trypanosoma cruzi* [10], [27], [37], [59], [65], [68], [85], [89], [92], [97], [112], [117], [120], [121], [124], [127], [135], [140], [149], and between chronic conditions of the genitourinary tract and *Chlamydia trachomatis* were the strongest in this data set [60], [73], [136]; at least three of Hill's criteria and at least three of Koch's postulates were fulfilled for those associations.

A more high-resolution analysis focusing on associations that are not well established for the five major disease areas (i.e. neoplasms, nervous system, respiratory system, circulatory system, and digestive system) is provided below.

#### Neoplasms

There were 31 studies identified in the study that examined associations between infectious agents and various neoplasms. Fifty-six individual associations linked carcinogenic viruses, parasites, and bacteria to 19 cancer types (i.e. on average 2·9 associations per cancer type, Figure 5). There were at least five publications describing associations between the different infectious agents and neoplasms with the exception of hepatitis E virus (HEV), *Chlamydia spp.*, and *Schistosoma spp.* There were only two publications identified that described associations between *Chlamydia spp.* and lung cancer, as well as two publications that described associations between *Schistosoma spp.* and bladder cancer or colonic polyposis. A negative association between HEV and liver cancer was reported in one publication.

Nine SRs, one RCT, 12 prospective cohort studies, four retrospective cohort studies, and five case-control studies were included. All associations except the association between HEV and liver cancer were described in at least one systematic review (NHMRC evidence level I). All associations were described in at least one prospective cohort study (NHMRC evidence level II), with the exception of the associations between *Chlamydia spp.* and lung cancer and *Schistosoma spp.* and bladder cancer or colonic polyposis where the reported study designs belonged to the NHMRC evidence level III.

There was a tendency towards a higher number of Hill's criteria being fulfilled compared to Koch's postulates, and Hill's sixth criterion (temporality) was most frequently fulfilled. With the exception of HEV and liver cancer, where a negative association was reported, at least two of Hill's criteria were fulfilled for the associations described. Two of Koch's postulates were fulfilled for all associations except HBV and hepatocellular carcinoma (HCC), HCV and HCC or cholangiocarcinoma, *Schistosoma* spp. and bladder cancer or colonic polyposis, and HEV and liver cancer.

When combining the three different methods for the assessment of the strength of the association three associations remained as weak: *Chlamydia spp.* and lung cancer, and *Schistosoma spp.* and bladder cancer or colonic polyposis.

#### Nervous system

A large number of infectious agents were implicated to play a role in the development of chronic conditions of the nervous system. There were 18 studies identified in the study that examined associations between infectious agents and chronic conditions of the nervous system. Forty-two associations in our systematic study linked infectious agents to 14 chronic conditions of the nervous system (i.e., 3·0 associations per chronic condition identified, Figure 5). For all associations between infectious agents and chronic conditions of the nervous system, there were not more than up to five publications identified. For the majority of associations (77%), there was only one publication identified. It should be noted that a negative association between EBV and multiple sclerosis was reported.

Five SRs, one RCT, four prospective cohort studies, three retrospective cohort studies, and five case-control studies were included. In 62%, the associations were described in at least one systematic review, prospective cohort study or RCT. The remaining associations were described in retrospective cohort studies or case-control studies.

Again, there was a tendency towards a higher number of Hill's criteria being fulfilled compared to Koch's postulates, and Hill's sixth criterion (temporality) was most frequently fulfilled. One to three of Hill's criteria were fulfilled in all but seven of the associations (27%); CMV and neuromyelitis optica, VZV and neuromyelitis optica, multiple sclerosis or Ramsay Hunt syndrome, *T. cruzi* and CNS impairment, *Rickettsia spp.* and chronic fatigue syndrome, and adenovirus, rotavirus, or RSV and multiple sclerosis. Two of Koch's postulates were not fulfilled in any of the associations; none of Koch's postulates were fulfilled in 23% and one postulate was fulfilled in 77%.

The following associations were defined as less established when the three different methods were applied to the data: *T. cruzi* and CNS impairment, *Rickettsia spp.* and chronic fatigue syndrome, and adenovirus, rotavirus, or RSV and multiple sclerosis.

#### Respiratory system

A large number of infectious agents were implicated to play a role in the development of chronic conditions of the respiratory system. There were 21 studies retrieved in the searches examining associations between infectious agents and chronic conditions of the respiratory system. Ninety-one associations described in the studies in our systematic study linked infectious agents to 12 chronic conditions of the respiratory system (i.e., on average 7·6 associations per chronic condition described, Figure 6). For all associations between infectious agents and chronic conditions of the respiratory system, there were five publications or less identified. For the majority of associations (74%), there was only one publication identified, while the other associations were supported by more than one study. It should be noted that for RSV and rhinovirus, negative associations were reported.

Seven SRs, zero RCTs, four prospective cohort studies, three retrospective cohort studies, and seven case-control studies were included. In 46% of the associations, at least one systematic review or prospective cohort study was identified, in the remaining associations, retrospective cohort studies or case-control studies (NHRMC evidence level III) described the associations.

The number of Hill's criteria and Koch's postulates were generally fulfilled to a similar extent. Koch's second postulate (isolated from diseased material) was most frequently fulfilled. Only one of Koch's postulates was fulfilled in 24% of all associations.

The following four associations were defined as less established and in need of further research when the three methods where combined; *Entamoeba coli* or *Giardia intestinalis* and chronic allergic rhinis, rhinovirus or RSV and asthma, and *Candida krusei* and chronic rhinosinusitis.

#### Circulatory system

Twenty-eight studies were identified that described associations between infectious agents and chronic conditions of the circulatory system. There were 37 associations described that linked viruses, bacteria, and parasites to 14 chronic conditions of the circulatory system (i.e., 2·6 associations per chronic condition described, Figure 7). Apart from the associations between *C. pneumoniae* and coronary heart disease or atherosclerosis and *T. cruzi* and cardiomyopathy or heart failure, where nine and ten publications were identified, respectively, there were one to three publications describing each of the associations between the infectious agents and chronic conditions of the circulatory system. It should be noted that a negative association was reported in one study for the associations between both *C. pneumoniae* and cytomegalovirus (CMV) and coronary heart disease.

Six SRs, one RCT, eight prospective cohort studies, four retrospective cohort studies, and nine case-control studies were included. In 44% of the associations, systematic reviews or prospective cohort studies were identified; 50% of the associations were described in retrospective cohort studies and the study describing the negative association between CMV and coronary heart disease was a case-control study.

Hill's sixth criterion (temporality) was most frequently fulfilled. One to three of Hill's criteria were fulfilled in all but three associations; CMV and coronary heart disease and HBV or HCV and myocardial fibrosis. At least two of Koch's postulates were fulfilled in four associations only (25%); in the remaining associations (75%), zero or one of Koch's postulates was fulfilled.

The combined result of the methods for assessing the strength of the individual associations resulted in that the associations between HIV, HBV or HCV and myocardial fibrosis as well as the association between CMV and coronary heart disease where defined as weak, requiring more research.

#### Digestive system

There were 44 studies identified examining associations between infectious agents and chronic conditions of the digestive system. A number of viruses, bacteria, and parasites were linked through 70 associations to 12 chronic conditions of the digestive system (i.e., 5·8 associations per chronic condition identified, Figure 7). Apart from the associations between *H. pylori*, HBV, and HCV and chronic conditions of the digestive system, which were described in more than 10 publications, each association was described in one or two publications. It should be noted that negative associations were reported for the associations between hepatitis G virus (HGV) and chronic liver disease and cirrhosis, HEV and cirrhosis, and *Giardia lamblia* and irritable bowel syndrome (IBS).

Five SRs, one RCT, 15 prospective cohort studies, 13 retrospective cohort studies, and 10 case-control studies were included. Associations with *Campylobacter jejuni*, hepatitis A virus (HAV), HGV, HIV, and *G. lamblia* were only described in retrospective cohort studies and case-control studies.

There was a tendency towards a higher number of Hill's criteria being fulfilled compared to Koch's postulates. One to three of Hill's criteria were fulfilled in all but four associations; between HIV and cirrhosis, transfusion-transmitted virus and chronic liver dysfunction, *G. lamblia* and IBS, and HEV and cirrhosis. Less than two of Koch's postulates were fulfilled in all but two associations; those between *H. pylori* and *Tropheryma whipplei* and diseases of the digestive system.

When applying the combination of the methods the association between HIV and cirrhosis, HAV and chronic liver disease, and *C. jejuni* and chronic gastroenteritis were found to be less established.

## Discussion

Over the last decades there has been a global shift towards higher chronic disease burden, relative to infectious diseases [1]. In Europe alone, with its ageing population, chronic conditions account for 86% of all deaths, 77% of the disease burden, and up to 80% of health care expenditures. Affordable and effective strategies to deal with this rising disease burden are an urgent priority in order to attenuate the adverse impact on public health. We evaluated the evidence describing associations between infectious agents and chronic conditions and assessed the causal inference of such associations. Five pathogens accounted for 60% of the associations documented in the literature; they were HIV, HCV, *H. pylori*, HBV, and *C. pneumoniae*. They were associated with 37 different chronic conditions. Thus, prevention and treatment of these five pathogens would be the most promising public health strategy to tackle chronic disease burden. By concentrating research efforts on a few promising areas, like the five pathogens identified here, the public health burden arising from chronic conditions can be reduced.

In this systematic study, few infectious agents were strongly linked to chronic conditions. There was a tendency towards a higher percentage of Hill's criteria being fulfilled compared to Koch's postulates; more than two of Koch's postulates were only fulfilled in exceptional cases. This may be due to the fact that Hill's criteria were developed for epidemiological studies while Koch's postulates were developed for experimental studies (often employing animal models, which were not included in the study) and the search strategy developed for the current study was designed to capture the former. In the data identified in this study, Hill's sixth criterion (temporality) and Koch's second postulate (presence in tissue) were fulfilled in the largest share of the associations identified. They constitute logical proofs of causation, regardless of whether the authors intentionally considered Hill's criteria and Koch's postulates while conducting the study.

There are certain difficulties associated with assessment of causality. The current models of causality work well for an infectious agent that is both necessary and sufficient for developing the condition, but it may be difficult to prove a causal relationship if an infectious agents is acting as a cofactor in the aetiology of a chronic condition. Also, certain infectious agents are ubiquitous in the population but are only capable of inflicting harm in certain instances, which may be determined by the timing and location of the infection, condition of the host, or environmental factors. Also, innocuous infectious agents may colonise the host after onset of disease and may, thus, be isolated from diseased tissue although they do not play a role in the aetiology. Other technical obstacles include difficulties in isolating and culturing certain infectious agents, it may be difficult or unethical to confirm the association in animal models or in humans, and the infectious agent may be cleared after the initial infection making it hard to identify the causative factor. Also, even with a great deal of data on the aetiologic nature of a chronic condition, the judgements are subject to interpretation.

A way to advance the current medical science within this field would be to link patient-level databases for infectious diseases with databases for chronic conditions, such as cancer registries in which it is compulsory for health care providers to register new cancer cases. Linkage analyses would enable longitudinal studies of the natural history of chronic conditions, particularly the preclinical phase, designed to establish new pathogen-disease associations or to examine the nature and timing of infections. Insights from such studies can lead public health interventions designed to avoid or minimise sequelae from chronic conditions. This could also generate measures for estimating the population attributable fraction (PAF), which is a valuable measure for determining the proportion of the chronic condition that may be caused by an infectious agent. In order to complement the assessment of the strength of the associations, an attempt was also made in this study to identify the proportions of the chronic conditions that are caused by infectious agents by extracting data on the PAF. However, only a handful of studies in this data set presented PAF estimates and it was, thus, not possible to conduct any comparative analyses on those estimates.

Many different disease areas and infectious agents were identified in this study and the analysis revealed that the majority of individual associations were described in only one to two studies each. Thus the area under investigation seems diverse and explorative rather than focused and validated. It may, therefore, be advisable to focus on the five promising pathogens discussed above and drive the development of preventive measures, such as vaccines or antimicrobials, for these. For a number of the chronic conditions identified in this study, there are already preventive strategies or interventions directed towards the infectious agents at hand. Such associations include *H. pylori* or HPV and neoplasms [19], [61], [72], [77], [85], [94], [106], [114], [147], *T. cruzi* and chronic conditions of the circulatory system [10], [27], [89], [92], [112], [117], [127], *C. trachomatis* and chronic conditions of the genitourinary system [60], and *H. pylori* or *T. whipplei* and chronic conditions of the digestive system [28], [54], [75], [77], [83], [90], [96], [102], [102], [106], [107], [123], [151,157,157].

On the contrary, for a number of chronic conditions, preventive strategies or interventions directed towards the infectious agents are currently not at hand. Such associations include *C. pneumoniae* and *Mycobacterium* spp. and chronic conditions of the circulatory system[37], [59], [65], [66], [68], [85], [97], [121], [124], [135]; *C. pneumoniae* and neoplasms [84], [85]; *H. pylori* and *D. folliculorum* and chronic conditions of the skin and subcutaneous tissue [53], [55], [96], adenovirus, influenza virus, parainfluenza virus, *Aspergillus* spp., *Candida* spp., *Chlamydia* spp., *H. influenzae*, *Klebsiella* spp., *Moraxella catarrhalis Mycobacterium* spp., *P. aeruginosa*, *Staphylococcus* spp., *Streptococcus spp.*, *Toxocara* spp., or *U. urealyticum* and chronic conditions of the respiratory system [13], [38], [45], [50], [57], [71], [74], [85], [93], [153]. For many of these conditions, treatment of the acute infection is warranted, regardless of the long-term consequences. Access to care is therefore a crucial aspect of prevention of chronic conditions in the long run. For these associations, further research, which ultimately may facilitate translation into healthcare interventions and prevention, is called for.

In contrast, for many infectious agents the link with chronic diseases was weak which therefore does not warrant translation into public health interventions. Continued research and novel methods are needed in order to enhance our knowledge base regarding these potential associations.

### Limitations

One of the limitations of this study is that some important infectious agents associated with significant chronic conditions may have been overlooked due to small numbers of publications or difficulties in assessing these agents because of technical limitations. For example, the results of this study revealed that certain well-established associations were not captured, such as the association between Human Herpes Virus-8 (HHV-8) and Kaposi's sarcoma and between EBV and various malignancies (e.g., Burkitt's lymphoma and nasopharyngeal carcinoma) [3]. Reasons for this may be traced back to the search strategy which included a search facet for ‘chronic conditions’ (i.e., diseases or illnesses, disabilities and sequelae lasting longer than three months). Malignant diseases such as Kaposi's sarcoma and nasopharyngeal carcinoma may rarely be referred to as ‘chronic diseases’ and this may be the reason to why these associations where not identified in the search. Moreover, all categories of infectious agents were included in this study (e.g., bacteria, viruses, parasites, fungi, and prions). Yet, none of the studies identified described chronic conditions associated with prions. Again, this may be due to the design of the search strategy. It is possible that the search terms included in the facet ‘Aetiology’ (e.g., terms related to infection, disease transmission, and host-pathogen interactions) or the ‘Study design’ facet restricted the searches, which may have led to exclusion of studies describing associations between prions and chronic conditions. The facets were designed and combined in order to achieve a search strategy with a good balance between sensitivity and precision; as the area examined was large, the search filters had to be wide enough to detect relevant studies but narrow enough to filter out non-relevant studies. In order to overcome the potential of publication bias, at least in part, we also included textbooks and non-systematic literature review papers in our study to capture any other papers the search might have missed. Nevertheless, few publications due to the recency of a discovery or lack of publications due to technical limitations, remain key limitations of many systematic reviews, including this study.

This study is also limited by the fact that standardised case definitions of infection states (i.e., initial infection, re-infection, active infection, persistent infection, latent infection, or co-infection) or chronic conditions are not available. Furthermore, some conditions are not directly caused by the infection (e.g. neoplasms and HIV) or might be the result of treatment (e.g. cardiovascular disease and HIV). Thus, there is a certain degree of heterogeneity in the analysed material. Moreover, this study focused on human studies and was, thus, somewhat limited to epidemiological studies, rather than including *in vitro* microbiological assessments or animal models.

### Conclusion and Future Outlook

The global public health burden associated with chronic conditions is significant. Infectious agents have been implicated in the aetiology of a number of these chronic conditions; for cancer alone, infectious agents are responsible for almost 22% of cancer deaths in low- and middle-income countries and 6% in industrialised countries [158], [159]. Our analysis identified a number of intervention entry points, specifically HIV, HCV, *H. pylori*, HBV, and *C. pneumoniae* as established contributors to the chronic disease burden. The diverse relationships between infectious agents and chronic conditions create a cascade of opportunities to reduce the impact of chronic conditions by interrupting the infection before irreversible consequences occur. An example of such an intervention option is universal voluntary HIV testing with immediate antiretroviral therapy as a strategy for elimination of HIV transmission [160]. While a thorough discussion of these intervention options is beyond the scope of this study, focusing on associations with a strong causal inference can help alleviate the global chronic disease burden.

## Supporting Information

Table S1 **Systematic searches run in MEDLINE® and Embase® in November 2010, via** www.embase.com **.**
(DOCX)

Table S2 **Systematic searches run in the Cochrane Reviews and Cochrane Clinical Trials databases in November 2010.**
(DOCX)

Table S3 **Overview of associations identified in cross-sectional studies, case studies/case series/case reports, literature reviews, and pathological assessment studies (i.e., NHMRC level IV studies).**
(DOCX)

Table S4 **Grouping of infectious agents into classes (i.e., bacteria, viruses, fungi, parasites).**
(DOCX)
