# Supplementary material for: Causal Inference Regarding Infectious Aetiology of Chronic Conditions: A Systematic Review
Source: PLoS One. 2013 Jul 25;8(7):e68861. doi: 10.1371/journal.pone.0068861 (PMC3723854; doi:10.1371/journal.pone.0068861)
Supplement: Table S1 — Systematic searches run in MEDLINE® and Embase® in November 2010, via www.embase.com . (DOCX) [file pone.0068861.s001.docx]

**Supporting information: SEARCH STRATEGY**

**Table 1: Systematic searches run in MEDLINE^®^ and Embase^®^ in November 2010, via** [**www.embase.com**](http://www.embase.com)

| **Facets** | **Search terms** |
| --- | --- |
| Chronic conditions (i.e., diseases, disabilities, and sequelae lasting at least three months) | ‘Chronic disease'/exp OR (chronic NEXT/1 diseas*) OR (chronic NEXT/1 illnes*) OR ‘Disability’/exp OR (chronic NEAR/3 disabilit*) OR (chronic NEAR/3 sequel*) OR ‘postpoliomyelitis syndrome’/de |
| AND | |
| Infection | ‘Infection’/exp OR (infectio*):ab,ti OR (infecti* NEXT/1 agent*) OR ‘communicable’ NEXT/1 (‘disease’ OR ‘diseases’) OR 'disease transmission'/exp OR disease* NEAR/2 transmission OR 'host-pathogen' NEXT/1 interaction* OR ((progressive NEAR/2 tissue) AND pathology) OR (organ NEAR/2 decompens*) |
| AND | |
| Aetiology | Etiology/exp OR (‘etiology’ OR ‘aetiology’):ab,ti OR (‘pathogenesis’):ab,ti OR causal* OR causation OR cause OR (‘koch’ OR (‘Hill’ AND ‘causation’)):ab,ti OR Attributable NEXT/1 (fraction OR risk OR proportion) |
| AND | |
| Study design | ‘Epidemiology'/exp OR (epidemi*):ab,ti OR 'epidemiological data'/exp OR 'epidemiological data' OR (inciden* OR prevalence OR mortalit* OR survival):ab,ti OR (geographic NEXT/1 distribution) OR (seasonal NEXT/1 variation) OR ‘cohort analysis'/exp OR ‘cohort' NEXT/1 ('study' OR 'studies') OR cohort*:ab,ti OR 'longitudinal study'/exp OR 'longitudinal' NEXT/1 ('study' OR 'studies') OR 'follow up'/exp OR ('follow up' OR ‘followup’) NEXT/1 (‘study’ or ‘studies’) OR ‘clinical study'/de OR ('clinical' NEXT/1 ('study' OR 'studies')):ab,ti OR ‘major clinical study’/exp OR 'major clinical' NEXT/1 ('study' OR 'studies') OR 'clinical trial'/exp OR (clinical NEXT/1 trial*):ab,ti OR 'intervention study'/exp OR 'intervention' NEXT/1 ('study' OR 'studies') OR 'retrospective study'/exp OR ‘prospective study’/exp OR ('retrospective’ OR ‘prospective’) NEXT/1 (‘study' OR ‘studies’) OR ‘crossover procedure’/exp OR (cross*over) NEXT/1 (study OR studies) OR ‘case control study’/exp OR 'case control' NEXT/1 ('study' OR 'studies') OR ‘case study’/exp OR ‘case report’/exp OR (case NEXT/1 (study OR studies OR report* OR serie*)) OR ‘review'/exp OR ‘review’ |
