# Supplementary material for: Causal Inference Regarding Infectious Aetiology of Chronic Conditions: A Systematic Review
Source: PLoS One. 2013 Jul 25;8(7):e68861. doi: 10.1371/journal.pone.0068861 (PMC3723854; doi:10.1371/journal.pone.0068861)
Supplement: Table S2 — Systematic searches run in the Cochrane Reviews and Cochrane Clinical Trials databases in November 2010. (DOCX) [file pone.0068861.s002.docx]

**Supporting Information: SEARCH STRATEGY**

**Table 2: Systematic searches run in the Cochrane Reviews and Cochrane Clinical Trials databases in November 2010**

| **Facets** | **Search terms** |
| --- | --- |
| Chronic conditions (i.e., diseases, disabilities, and sequelae lasting at least three months) | MeSH descriptor Chronic Disease explode all trees OR (chronic NEXT diseas*) OR (chronic NEXT illnes*) OR (chronic NEAR/3 disabilit*) OR (chronic NEAR/3 sequel*) OR MeSH descriptor Postpoliomyelitis Syndrome explode all trees |
| AND | |
| Infection | MeSH descriptor Infection explode all trees OR (infectio*):ab,ti OR (infecti* NEXT agent*) OR MeSH descriptor Communicable Diseases explode all trees OR communicable NEXT (disease OR diseases) OR MeSH descriptor Disease Transmission, Infectious explode all trees OR disease* NEAR/2 transmission OR MeSH descriptor Host-Pathogen Interactions explode all trees OR host-pathogen NEXT interaction* OR ((progressive NEAR/2 tissue) AND pathology) OR (organ NEAR/2 decompens*) |
| AND | |
| Aetiology | MeSH descriptor Causality explode all trees OR (etiology OR aetiology):ab,ti OR (pathogenesis):ab,ti OR causal* OR causation OR cause OR (koch OR (Hill AND causation)):ab,ti OR Attributable NEXT (fraction OR risk OR proportion) |
| AND | |
| Study design | MeSH descriptor Epidemiology explode all trees OR (epidemi*):ab,ti OR MeSH descriptor Incidence explode all trees OR MeSH descriptor Prevalence explode all trees OR MeSH descriptor Mortality explode all trees OR (inciden* OR prevalence OR mortalit* OR survival):ab,ti OR (geographic NEXT distribution) OR (seasonal NEXT variation) OR MeSH descriptor Epidemiologic Studies explode all trees OR Epidemiologic NEXT (study or studies) OR cohort*:ab,ti OR Case NEXT control NEXT (study OR studies) OR MeSH descriptor Cross-Over Studies explode all trees OR (cross*over) NEXT (study OR studies) OR longitudinal NEXT (study OR studies) OR ((follow NEXT up) OR followup) NEXT (study OR studies) OR (retrospective OR prospective) NEXT (study OR studies) OR MeSH descriptor Intervention Studies explode all trees OR intervention NEXT (study OR studies)  (clinical NEXT (study OR studies)):ab,ti OR (major NEXT clinical) NEXT (study OR studies) OR (clinical NEXT trial*):ab,ti |
