# Supplementary material for: Causal Inference Regarding Infectious Aetiology of Chronic Conditions: A Systematic Review
Source: PLoS One. 2013 Jul 25;8(7):e68861. doi: 10.1371/journal.pone.0068861 (PMC3723854; doi:10.1371/journal.pone.0068861)
Supplement: Table S3 — Overview of associations identified in cross-sectional studies, case studies/case series/case reports, literature reviews, and pathological assessment studies (i.e., NHMRC level IV studies). (DOCX) [file pone.0068861.s003.docx]

**Supporting Information Table 3: OVERVIEW OF ASSOCIATIONS IDENTIFIED IN CROSS-SECTIONAL STUDIES, CASE STUDIES/CASE SERIES/CASE REPORTS, LITERATURE REVIEWS, AND PATHOLOGICAL ASSESSMENT STUDIES**

**Table 3: Overview of associations identified in cross-sectional studies, case studies/case series/case reports, literature reviews, and pathological assessment studies (i.e., NHMRC level IV studies)**

| **Disease area** | **Infectious agent** | **Number of NHMRC level IV publications** | **Number of Koch’s postulates fulfilled** | **Number of Hill’s criteria fulfilled** |
| --- | --- | --- | --- | --- |
| Neoplasms | *Aggregatibacter actinomycetemcomitans* | 1 | 1 | 0 |
|  | *Aspergillus spp.* | 2 | 1 | 1 |
|  | *Bartonella* spp. | 1 | 2 | 1 |
|  | BK virus | 2 | 1 | 2 |
|  | *Borrelia burgdorferi* | 1 | 1 | 2 |
|  | *Candida albicans* | 1 | 0 | 2 |
|  | *Chlamydia spp.* | 5 | 2 | 4 |
|  | *Chlonorchis sinensis* | 3 | 0 | 1 |
|  | *Clostridium septicum* | 1 | 0 | 0 |
|  | *Cryptococcus neoformans* | 1 | 1 | 1 |
|  | Cytomegalovirus | 3 | 1 | 2 |
|  | Epstein-Barr virus | 25 | 2 | 3 |
|  | *Fusarium spp.* | 1 | 0 | 1 |
|  | *Helicobacter spp.****** | 33 | 3 | 3 |
|  | Hepatitis B virus | 33 | 3 | 4 |
|  | Hepatitis C virus | 41 | 3 | 4 |
|  | Hepatitis D virus | 2 | 0 | 1 |
|  | Herpes simplex virus | 1 | 0 | 1 |
|  | Human endogenous retroviruses | 2 | 2 | 2 |
|  | Human foamy virus | 1 | 1 | 0 |
|  | Human herpesvirus-6 | 3 | 1 | 1 |
|  | Human herpesvirus-8***** | 18 | 2 | 3 |
|  | Human immunodeficiency virus | 17 | 3 | 3 |
|  | Human papillomavirus | 27 | 2 | 3 |
|  | Human T-lymphotropic virus | 12 | 1 | 3 |
|  | Jaagsiekte sheep retrovirus | 1 | 1 | 0 |
|  | John Cunningham virus | 2 | 0 | 2 |
|  | Liver flukes | 1 | 0 | 1 |
|  | Mouse mammary tumour virus | 1 | 2 | 0 |
|  | *Mycoplasma* spp. | 1 | 1 | 3 |
|  | *Opisthorchis viverrini* | 3 | 0 | 1 |
|  | *Schistosoma* spp. | 8 | 1 | 3 |
|  | SEN virus***** | 1 | 0 | 0 |
|  | Simian retrovirus-3 | 1 | 2 | 0 |
|  | Simian vacuolating virus 40 | 3 | 3 | 2 |
|  | *Strongyloides stercoralis* | 1 | 0 | 1 |
|  | Torque teno virus | 1 | 0 | 1 |
|  | Transfusion transmitted virus***** | 1 | 0 | 0 |
|  | *Treponema pallidum* | 1 | 0 | 1 |
|  | Xenotropic murine leukaemia virus-related virus | 1 | 1 | 0 |
| Blood and blood-forming organs | *Brucella spp.* | 1 | 1 | 1 |
|  | *Chlamydia pneumoniae* | 1 | 0 | 0 |
|  | *Coxiella burnetii* | 1 | 0 | 0 |
|  | Cytomegalovirus | 2 | 1 | 1 |
|  | Epstein-Barr virus | 3 | 1 | 2 |
|  | *Escherichia coli* | 5 | 1 | 2 |
|  | *Granulibacter bethesdensis* | 1 | 3 | 1 |
|  | *Helicobacter pylori* | 4 | 3 | 3 |
|  | Hepatitis B virus | 3 | 1 | 2 |
|  | Hepatitis C virus | 9 | 2 | 4 |
|  | Hookworms | 2 | 0 | 1 |
|  | Human immunodeficiency virus | 3 | 1 | 2 |
|  | Influenza virus | 1 | 0 | 0 |
|  | Intracisternal A-type particles | 1 | 1 | 0 |
|  | *Leishmania donovani* | 1 | 0 | 1 |
|  | Measles virus | 1 | 0 | 0 |
|  | Mumps virus | 1 | 0 | 0 |
|  | *Mycobacterium spp.* | 2 | 1 | 1 |
|  | *Mycoplasma pneumoniae* | 1 | 0 | 0 |
|  | Nematodes | 1 | 0 | 1 |
|  | Parainfluenza virus | 1 | 0 | 0 |
|  | Parvovirus B19 | 8 | 2 | 2 |
|  | Respiratory syncytial virus | 1 | 0 | 0 |
|  | *Schistosoma* spp. | 2 | 0 | 2 |
|  | *Serratia marcescens* | 1 | 0 | 0 |
|  | *Staphylococcus aureus* | 1 | 0 | 0 |
|  | *Trichuris trichiura* | 2 | 1 | 2 |
|  | Varicella zoster virus | 1 | 0 | 0 |
| Endocrine, nutritional, and metabolic disorders | Adenovirus-36 | 1 | 4 | 2 |
|  | *Aggregatibacter actinomycetemcomitans* | 2 | 0 | 1 |
|  | *Ascaris lumbricoides* | 1 | 0 | 2 |
|  | *Bacteroides forsythus* | 1 | 0 | 1 |
|  | *Campylobacter rectus* | 1 | 0 | 1 |
|  | *Capillaria philippinensis* | 1 | 0 | 1 |
|  | *Chlamydia pneumoniae* | 2 | 1 | 1 |
|  | Coxsackievirus | 10 | 3 | 3 |
|  | *Cryptosporidium parvum* | 1 | 0 | 1 |
|  | Cytomegalovirus | 2 | 3 | 1 |
|  | Echovirus | 1 | 1 | 1 |
|  | *Escherichia coli* | 1 | 0 | 2 |
|  | *Fusobacterium nucleatum* | 1 | 0 | 1 |
|  | *Giardia intestinalis* | 1 | 0 | 1 |
|  | *Helicobacter pylori* | 2 | 0 | 1 |
|  | Hepatitis C virus | 11 | 1 | 2 |
|  | Hookworm | 1 | 0 | 1 |
|  | Human endogenous retroviruses | 1 | 2 | 1 |
|  | Human foamy virus | 1 | 1 | 0 |
|  | Human immunodeficiency virus | 4 | 1 | 3 |
|  | Intracisternal A-type particles | 1 | 1 | 0 |
|  | *Isospora belli* | 1 | 0 | 2 |
|  | Mumps virus | 1 | 0 | 0 |
|  | Nematodes | 1 | 0 | 1 |
|  | *Porphyromonas gingivalis* | 3 | 0 | 1 |
|  | Rotavirus | 1 | 2 | 1 |
|  | Rubella virus | 2 | 2 | 1 |
|  | *Schistosoma* spp. | 2 | 0 | 2 |
|  | SMAM-1 virus | 1 | 1 | 1 |
|  | *Strongyloides stercoralis* | 1 | 0 | 1 |
|  | *Tanerella forsythia* | 1 | 0 | 0 |
|  | *Toxoplasma gondii* | 1 | 0 | 1 |
|  | *Treponema denticola* | 2 | 0 | 1 |
|  | *Yersinia enterolytica* | 1 | 0 | 0 |
| Mental and behavioural disorders | Adenovirus | 1 | 0 | 1 |
|  | *Aggregatibacter actinomycetemcomitans* | 1 | 0 | 2 |
|  | *Alloiococcus otitidis* | 1 | 0 | 1 |
|  | *Ankylostoma duodenale* | 1 | 0 | 0 |
|  | *Bartonella spp.* | 1 | 1 | 1 |
|  | Borna virus | 5 | 2 | 2 |
|  | *Borrelia spp.* | 4 | 2 | 3 |
|  | *Chlamydia pneumoniae* | 7 | 2 | 1 |
|  | Cytomegalovirus | 3 | 0 | 1 |
|  | Epstein-Barr virus | 1 | 0 | 1 |
|  | *Haemophilus influenzae* | 2 | 0 | 1 |
|  | Herpes simplex virus | 6 | 0 | 2 |
|  | Human endogenous retroviruses | 2 | 2 | 1 |
|  | Human herpes virus-6 | 2 | 2 | 1 |
|  | Human immunodeficiency virus | 6 | 2 | 3 |
|  | Influenza virus | 3 | 0 | 1 |
|  | *Listeria monocytogenes* | 1 | 0 | 0 |
|  | Measles virus | 2 | 1 | 0 |
|  | *Moraxella catarrhalis* | 1 | 0 | 1 |
|  | *Mycoplasma spp.* | 1 | 2 | 1 |
|  | *Necator americanus* | 1 | 0 | 0 |
|  | Neisseria meningitidis | 1 | 0 | 0 |
|  | Parainfluenza virus | 1 | 0 | 1 |
|  | *Porphyromonas gingivalis* | 1 | 0 | 2 |
|  | Respiratory syncytial virus | 1 | 0 | 1 |
|  | Rhinovirus | 1 | 0 | 1 |
|  | Rubella virus | 2 | 0 | 1 |
|  | *Salmonella typhi* | 1 | 0 | 2 |
|  | *Schistosoma* spp. | 2 | 0 | 1 |
|  | *Staphylococcis aureus* | 1 | 0 | 1 |
|  | *Streptococcus* spp. | 8 | 0 | 1 |
|  | *Taenia solium* | 2 | 0 | 1 |
|  | *Toxoplasma gondii* | 6 | 2 | 2 |
|  | *Treponema spp.* | 2 | 2 | 2 |
|  | *Trichuris trichiura* | 1 | 0 | 0 |
|  | Varicella zoster virus | 2 | 0 | 1 |
| Nervous system | *Actinomyces israelii* | 1 | 1 | 1 |
|  | Adenovirus | 2 | 2 | 1 |
|  | *Alliococcus otitidis* | 1 | 0 | 1 |
|  | *Blastocystis hominis* | 1 | 1 | 0 |
|  | Borna virus | 1 | 0 | 1 |
|  | *Borrelia burgdorferi* | 8 | 2 | 2 |
|  | *Brucella spp.* | 2 | 1 | 2 |
|  | *Campylobacter jejuni* | 8 | 2 | 3 |
|  | *Candida albicans* | 1 | 0 | 1 |
|  | Canine distemper virus | 1 | 0 | 0 |
|  | Chimpanzee cytomegalovirus | 1 | 0 | 0 |
|  | *Chlamydia pneumoniae* | 8 | 2 | 2 |
|  | Corona virus | 2 | 0 | 0 |
|  | *Coxiella burnetii* | 6 | 1 | 3 |
|  | Coxsackievirus | 1 | 0 | 1 |
|  | *Cryptococcus neoformans* | 2 | 1 | 2 |
|  | Cytomegalovirus | 13 | 1 | 3 |
|  | Echovirus | 1 | 0 | 0 |
|  | Epstein-Barr virus | 18 | 2 | 3 |
|  | *Gardnerella vaginalis* | 1 | 0 | 0 |
|  | *Haemophilus influenzae* | 3 | 1 | 1 |
|  | *Helicobacter pylori* | 3 | 0 | 1 |
|  | Herpes simplex virus | 10 | 2 | 3 |
|  | Human endogenous retroviruses | 3 | 3 | 3 |
|  | Human herpesvirus, unspecified | 1 | 0 | 0 |
|  | Human herpes virus-6 | 7 | 2 | 3 |
|  | Human herpes virus-7 | 1 | 0 | 0 |
|  | Human herpes virus-8 | 1 | 0 | 0 |
|  | Human immunodeficiency virus | 9 | 1 | 3 |
|  | Human papillomavirus | 1 | 0 | 1 |
|  | Human T-lymphotropic virus | 10 | 1 | 3 |
|  | Influenza virus | 8 | 2 | 1 |
|  | JHK retrovirus | 1 | 1 | 0 |
|  | *Listeria monocytogenes* | 1 | 1 | 2 |
|  | Marek's disease virus | 1 | 0 | 0 |
|  | Measles virus | 8 | 1 | 1 |
|  | *Mobiluncus spp.* | 1 | 0 | 0 |
|  | Moraxella catarrhalis | 2 | 0 | 1 |
|  | MS-associated agent | 1 | 0 | 0 |
|  | MS-associated retrovirus | 1 | 0 | 0 |
|  | Mumps virus | 2 | 1 | 1 |
|  | *Mycoplasma* spp. | 6 | 2 | 3 |
|  | Nipah virus | 1 | 0 | 1 |
|  | *Paragonimus* spp. | 1 | 0 | 1 |
|  | Parainfluenza virus | 5 | 1 | 2 |
|  | Paramyxovirus | 2 | 0 | 0 |
|  | Poliovirus | 13 | 2 | 2 |
|  | *Prevotella spp.* | 1 | 0 | 0 |
|  | Prions | 1 | 0 | 1 |
|  | Rabies virus | 2 | 0 | 0 |
|  | Respiratory syncytial virus | 2 | 2 | 1 |
|  | Retrovirus | 1 | 0 | 0 |
|  | Rhinovirus | 2 | 1 | 1 |
|  | Rubella virus | 4 | 1 | 1 |
|  | *Schistosoma* spp. | 3 | 1 | 2 |
|  | Scrapie agent | 1 | 0 | 0 |
|  | Simian virus 5 | 1 | 0 | 0 |
|  | SMON-like virus | 1 | 0 | 0 |
|  | *Staphylococcus aureus* | 3 | 1 | 1 |
|  | *Streptococcus* spp. | 4 | 1 | 1 |
|  | *Taenia solium* | 3 | 0 | 1 |
|  | Tick-borne encephalitis virus | 1 | 0 | 0 |
|  | *Toxoplasma gondii* | 2 | 0 | 2 |
|  | *Treponema pallidum* | 2 | 1 | 1 |
|  | *Ureaplasma urealyticum* | 1 | 0 | 0 |
|  | Varicella zoster virus | 8 | 2 | 3 |
|  | West Nile virus | 3 | 1 | 1 |
|  | *Wuchereria bancrofti* | 3 | 2 | 1 |
|  | *Yersinia enterocolitica* | 1 | 0 | 1 |
| Respiratory system | *Absidia corymbifera* | 1 | 0 | 1 |
|  | *Achromobacter xylosoxidans* | 1 | 0 | 1 |
|  | Adenovirus | 4 | 2 | 1 |
|  | *Alternaria spp.* | 1 | 1 | 0 |
|  | *Aspergillus spp.* | 11 | 3 | 3 |
|  | *Bacteroides fragilis* | 2 | 2 | 1 |
|  | *Bipolaris spicifera* | 2 | 1 | 2 |
|  | *Bordetella pertussis* | 4 | 2 | 2 |
|  | *Burkholderia cepacia* | 1 | 0 | 1 |
|  | *Candida spp.* | 1 | 2 | 1 |
|  | *Chlamydia spp.* ***** | 10 | 2 | 2 |
|  | *Cladosporium herbarum* | 1 | 1 | 0 |
|  | *Coxiella burnetii* | 2 | 1 | 1 |
|  | *Cryptostroma corticale* | 1 | 0 | 1 |
|  | *Curvularia lunata* | 2 | 1 | 2 |
|  | Cytomegalovirus | 4 | 2 | 1 |
|  | *Enterobacter spp.* | 2 | 1 | 2 |
|  | Epstein-Barr virus | 1 | 0 | 1 |
|  | *Escherichia coli* | 6 | 2 | 1 |
|  | *Eurotium amstelodami* | 1 | 0 | 1 |
|  | *Exserohilum rostratum* | 1 | 1 | 2 |
|  | Filarial nematodes | 1 | 1 | 1 |
|  | *Fusobacterium nucleatum* | 2 | 1 | 1 |
|  | *Haemophilus influenzae* | 20 | 2 | 4 |
|  | *Helicobacter pylori****** | 6 | 2 | 2 |
|  | Hepatitis A virus | 1 | 1 | 0 |
|  | Hepatitis B virus | 2 | 1 | 1 |
|  | Hepatitis C virus | 2 | 0 | 1 |
|  | Herpes simplex virus | 1 | 1 | 0 |
|  | Human endogenous retroviruses | 1 | 0 | 0 |
|  | Human immunodeficiency virus | 3 | 2 | 2 |
|  | Human papillomavirus | 1 | 0 | 1 |
|  | Influenza virus | 1 | 2 | 2 |
|  | *Klebsiella* spp. | 5 | 2 | 2 |
|  | Measles virus | 1 | 0 | 1 |
|  | *Moraxella catarrhalis* | 12 | 2 | 3 |
|  | *Mycobacterium spp.* | 4 | 1 | 2 |
|  | *Mycoplasma spp.****** | 6 | 2 | 2 |
|  | *Paragonimus spp.* | 1 | 0 | 1 |
|  | Parainfluenza virus | 2 | 2 | 2 |
|  | *Penicillium spp.* | 2 | 2 | 1 |
|  | *Pneumocystis jiroveci* | 1 | 1 | 0 |
|  | *Prevotella intermedia* | 2 | 1 | 1 |
|  | *Proprionibacterium* acnes | 3 | 1 | 2 |
|  | *Proteus mirabilis* | 3 | 2 | 1 |
|  | *Pseudallescheria boydii* | 1 | 0 | 2 |
|  | *Pseudomonas aeruginosa* | 13 | 3 | 3 |
|  | Respiratory syncytial virus | 7 | 2 | 4 |
|  | Rhinovirus | 2 | 0 | 1 |
|  | *Saccharopolyspora rectivirgula* | 1 | 0 | 1 |
|  | *Schistosoma* spp. | 2 | 0 | 2 |
|  | *Schizophillum commune* | 1 | 1 | 2 |
|  | *Serratia marcescens* | 1 | 1 | 1 |
|  | *Staphylococcus* spp. | 22 | 2 | 4 |
|  | *Stenotrophomonas maltophilia* | 1 | 0 | 1 |
|  | *Streptococcus* spp. | 17 | 3 | 4 |
|  | *Thermoactinomyces spp.* | 1 | 0 | 1 |
|  | *Trichophyton spp.* | 1 | 0 | 1 |
|  | *Trichosporon spp.* | 1 | 0 | 1 |
|  | *Ureaplasma urealyticum* | 1 | 2 | 3 |
|  | Variola virus | 1 | 0 | 2 |
| Eye and adnexa | Adenovirus | 1 | 0 | 1 |
|  | *Alcaligenes xylosoxidans* | 1 | 1 | 1 |
|  | *Bartonella* spp. | 1 | 1 | 1 |
|  | *Borrelia burgdorferi* | 1 | 0 | 1 |
|  | *Candida parapsilosis* | 1 | 0 | 1 |
|  | *Chlamydia trachomatis* | 4 | 2 | 2 |
|  | *Cryptococcus neoformans* | 1 | 0 | 1 |
|  | Cytomegalovirus | 3 | 1 | 3 |
|  | Epstein-Barr virus | 1 | 1 | 1 |
|  | Hepatitis C virus | 1 | 1 | 1 |
|  | Herpes simplex virus | 2 | 0 | 1 |
|  | Measles virus | 1 | 0 | 1 |
|  | *Mycobacterium tuberculosis* | 1 | 1 | 1 |
|  | *Nocardia asteroides* | 1 | 1 | 1 |
|  | *Onchocerca volvulus* | 2 | 0 | 2 |
|  | *Paragonimus* spp. | 1 | 0 | 1 |
|  | *Proprionibacterium acnes* | 1 | 0 | 1 |
|  | *Rhizobium radiobacter* | 1 | 1 | 2 |
|  | *Staphylococcus epidermidis* | 1 | 0 | 1 |
|  | *Taenia solium* | 1 | 0 | 1 |
|  | *Toxoplasma gondii* | 1 | 0 | 1 |
|  | *Tropheryma whipplei* | 1 | 0 | 1 |
|  | Vaccinia virus | 1 | 0 | 1 |
| Ear and mastoid process | Adenovirus | 1 | 0 | 1 |
|  | *Alloiococcus otitidis* | 1 | 0 | 1 |
|  | *Borrelia burgdorferi* | 1 | 0 | 1 |
|  | *Brucella melitensis* | 1 | 1 | 2 |
|  | Cytomegalovirus | 2 | 1 | 3 |
|  | *Haemophilus influenzae* | 4 | 0 | 2 |
|  | Human herpes virus | 1 | 0 | 1 |
|  | Influenza virus | 1 | 0 | 1 |
|  | Measles virus | 1 | 0 | 1 |
|  | *Moraxella catarrhalis* | 2 | 0 | 1 |
|  | Mumps virus | 1 | 0 | 1 |
|  | *Neisseria meningitidis* | 1 | 0 | 1 |
|  | Parainfluenza virus | 1 | 0 | 1 |
|  | *Proteus mirabilis* | 1 | 1 | 1 |
|  | Respiratory syncytial virus | 1 | 0 | 1 |
|  | Rhinovirus | 1 | 0 | 1 |
|  | Rubella virus | 1 | 0 | 1 |
|  | *Scedosporium apiospermum* | 1 | 1 | 2 |
|  | *Staphylococcus aureus* | 2 | 0 | 1 |
|  | *Streptococcus* spp. | 3 | 0 | 2 |
|  | *Treponema pallidum* | 1 | 0 | 1 |
|  | Vaccinia virus | 1 | 0 | 1 |
| Circulatory system | Adenovirus | 2 | 0 | 0 |
|  | *Aggregatibacter actinomycetemcomitans* | 6 | 1 | 2 |
|  | *Ascaris lumbricoides* | 1 | 0 | 2 |
|  | *Bacillus typhosus* | 1 | 0 | 0 |
|  | *Bacteroides forsythus* | 4 | 1 | 2 |
|  | *Borrelia spp.* | 4 | 1 | 1 |
|  | *Brucella spp.* | 1 | 1 | 1 |
|  | *Campylobacter rectus* | 1 | 0 | 1 |
|  | *Chlamydia pneumoniae* | 31 | 4 | 4 |
|  | *Coxiella burnetii* | 9 | 1 | 3 |
|  | Coxsackievirus spp. | 12 | 4 | 3 |
|  | Cytomegalovirus | 19 | 4 | 3 |
|  | Enterovirus | 1 | 0 | 0 |
|  | Epstein-Barr virus | 4 | 1 | 1 |
|  | *Escherichia coli* | 1 | 0 | 0 |
|  | Filarial nematodes | 3 | 1 | 1 |
|  | *Fusobacterium nucleatum* | 3 | 0 | 1 |
|  | *Giardia lamblia* | 1 | 0 | 2 |
|  | *Haemophilus influenzae* | 2 | 0 | 0 |
|  | *Helicobacter pylori* | 16 | 2 | 2 |
|  | Hepatitis A virus | 3 | 0 | 0 |
|  | Hepatitis C virus | 1 | 1 | 1 |
|  | Herpes simplex virus | 6 | 2 | 1 |
|  | *Histoplasma capsulatum* | 1 | 0 | 1 |
|  | Human immunodeficiency virus | 11 | 3 | 3 |
|  | Influenza virus | 5 | 1 | 3 |
|  | *Loa loa* | 1 | 0 | 0 |
|  | Marek's disease virus | 2 | 0 | 0 |
|  | Measles virus | 2 | 0 | 1 |
|  | *Mycobacterium spp.* | 5 | 2 | 3 |
|  | *Mycoplasma* spp. | 3 | 2 | 3 |
|  | *Porphyromonas gingivalis* | 12 | 3 | 2 |
|  | *Prevotella intermedia* | 3 | 1 | 2 |
|  | Ross River virus | 1 | 0 | 0 |
|  | Rubella virus | 1 | 0 | 1 |
|  | *Salmonella spp.* | 3 | 1 | 2 |
|  | *Staphylococcus spp.* | 1 | 1 | 3 |
|  | *Streptococcus* spp. | 10 | 2 | 3 |
|  | *Tannerella forsythia* | 2 | 0 | 0 |
|  | *Toxoplasma gondii* | 1 | 0 | 1 |
|  | *Treponema spp.* | 3 | 1 | 1 |
|  | *Trichinella spiralis* | 1 | 0 | 1 |
|  | *Trypanosoma cruzi* | 24 | 4 | 4 |
|  | *Wuchereria bancrofti* | 2 | 2 | 1 |
| Digestive system | Adenovirus | 2 | 1 | 1 |
|  | *Aeromonas hydrophila* | 1 | 1 | 1 |
|  | *Ascaris lumbricoides* | 1 | 0 | 1 |
|  | *Aspergillus flavus* | 1 | 1 | 0 |
|  | Astrovirus | 1 | 0 | 1 |
|  | *Blastocystis hominis* | 2 | 1 | 1 |
|  | *Borrelia spp.* | 1 | 1 | 1 |
|  | *Campylobacter jejuni* | 2 | 1 | 1 |
|  | *Candida albicans* | 1 | 0 | 2 |
|  | *Chlamydia pneumoniae* | 1 | 1 | 1 |
|  | *Clostridium difficile* | 2 | 2 | 1 |
|  | Coxsackievirus B | 1 | 3 | 2 |
|  | *Cryptosporidium parvum* | 1 | 0 | 1 |
|  | *Cyclospora cayetanensis* | 1 | 0 | 1 |
|  | Cytomegalovirus | 3 | 2 | 2 |
|  | *Echinococcus* spp. | 1 | 0 | 1 |
|  | *Entamoeba histolytica* | 1 | 1 | 1 |
|  | Epstein-Barr virus | 4 | 1 | 1 |
|  | *Escherichia coli* | 5 | 2 | 2 |
|  | *Fusobacterium nucleatum* | 1 | 0 | 1 |
|  | *Giardia lamblia* | 2 | 1 | 1 |
|  | *Helicobacter* spp.***** | 47 | 4 | 4 |
|  | Hepatitis A virus | 1 | 1 | 1 |
|  | Hepatitis B virus | 24 | 2 | 3 |
|  | Hepatitis C virus | 34 | 2 | 3 |
|  | Hepatitis D virus | 1 | 0 | 1 |
|  | Hepatitis E virus | 1 | 1 | 1 |
|  | Herpes simplex virus | 1 | 0 | 2 |
|  | Hookworms | 1 | 0 | 1 |
|  | Human immunodeficiency virus | 4 | 1 | 1 |
|  | Intracisternal A-type particles | 1 | 1 | 0 |
|  | *Isospora belli* | 1 | 0 | 1 |
|  | *Listeria monocytogenes* | 2 | 1 | 1 |
|  | Liver flukes | 1 | 0 | 1 |
|  | Measles virus***** | 3 | 1 | 1 |
|  | Microsporidia | 1 | 0 | 1 |
|  | Mouse mammary tumour virus | 1 | 1 | 1 |
|  | Mumps virus | 1 | 1 | 1 |
|  | *Mycobacterium spp.* | 9 | 2 | 2 |
|  | *Mycoplasma pneumoniae* | 1 | 0 | 0 |
|  | Nematodes | 1 | 0 | 1 |
|  | *Novosphingobium aromaticivorans* | 1 | 2 | 2 |
|  | *Peptostreptococcus micros* | 1 | 0 | 1 |
|  | *Prevotella melaninogenica* | 1 | 0 | 1 |
|  | Rotavirus | 2 | 0 | 1 |
|  | *Saccharomyces cerevisiae* | 1 | 1 | 1 |
|  | *Salmonella typhii* | 1 | 1 | 1 |
|  | *Schistosoma* spp. | 4 | 0 | 1 |
|  | *Staphylococcus aureus* | 1 | 0 | 1 |
|  | *Streptococcus pyogenes* | 1 | 0 | 1 |
|  | *Tropheryma whipplei* | 9 | 2 | 4 |
|  | *Trypanosoma cruzi* | 5 | 3 | 2 |
|  | *Yersinia enterocolitica* | 1 | 1 | 1 |
| Skin and connective tissue | *Acinetobacter baumannii* | 1 | 1 | 0 |
|  | *Alternaria alternata* | 1 | 1 | 0 |
|  | *Aspergillus fumigatus* | 1 | 1 | 0 |
|  | *Borrelia* spp. | 8 | 2 | 3 |
|  | *Chlamydia pneumoniae* | 1 | 2 | 2 |
|  | *Citrobacter freundii* | 1 | 1 | 0 |
|  | *Enterobacter cloacae* | 1 | 1 | 0 |
|  | *Enterococcus spp.* | 1 | 1 | 0 |
|  | *Escherichia coli* | 1 | 1 | 0 |
|  | *Helicobacter pylori* | 1 | 2 | 4 |
|  | Hepatitis C virus | 1 | 1 | 1 |
|  | Human endogenous retroviruses | 1 | 1 | 0 |
|  | Human herpesvirus-8***** | 1 | 0 | 0 |
|  | Human immunodeficiency virus | 1 | 1 | 1 |
|  | Intracisternal A-type particles | 1 | 1 | 0 |
|  | *Klebsiella spp.* | 1 | 1 | 0 |
|  | *Morganella morganii* | 1 | 1 | 0 |
|  | Murine leukaemia virus | 1 | 1 | 0 |
|  | *Mycobacterium intermedium* | 1 | 1 | 2 |
|  | *Nocardia asteroides* | 1 | 1 | 1 |
|  | *Pityrosporum ovale* | 1 | 0 | 0 |
|  | *Proteus mirabilis* | 1 | 1 | 0 |
|  | *Providencia stuartii* | 1 | 1 | 0 |
|  | *Pseudomonas aeruginosa* | 3 | 1 | 2 |
|  | *Schistosoma haematobium* | 1 | 0 | 1 |
|  | *Serratia marcescens* | 1 | 1 | 0 |
|  | *Staphylococcus* spp. | 8 | 1 | 3 |
|  | *Streptococcus pyogenes* | 3 | 1 | 1 |
|  | *Trichophyton spp.* | 2 | 1 | 0 |
|  | Vaccinia virus | 1 | 0 | 1 |
| Musculoskeletal system | Adenovirus | 1 | 0 | 1 |
|  | *Bartonella* spp. | 1 | 0 | 0 |
|  | *Blastomyces dermatitidis* | 1 | 0 | 0 |
|  | *Bordetella spp.* | 1 | 0 | 1 |
|  | *Borrelia spp.* | 19 | 1 | 3 |
|  | *Brucella spp.* | 2 | 1 | 1 |
|  | *Campylobacter* spp. | 7 | 0 | 1 |
|  | *Candida spp.* | 2 | 1 | 0 |
|  | Chikungunya virus | 1 | 0 | 1 |
|  | *Chlamydia spp.* | 11 | 2 | 3 |
|  | *Clostridium difficile* | 2 | 0 | 1 |
|  | *Coccidioides immitis/posadasii complex* | 1 | 0 | 0 |
|  | *Coxiella burnetii* | 1 | 0 | 0 |
|  | Coxsackievirus | 1 | 2 | 1 |
|  | Cytomegalovirus | 5 | 1 | 1 |
|  | *Echinococcus* spp. | 1 | 0 | 1 |
|  | *Eikenella corrodens* | 1 | 0 | 0 |
|  | Epstein-Barr virus | 9 | 2 | 1 |
|  | *Escherichia coli* | 2 | 1 | 1 |
|  | *Haemophilus influenzae* | 2 | 0 | 1 |
|  | *Helicobacter pylori* | 2 | 0 | 2 |
|  | Hepatitis B virus | 4 | 0 | 1 |
|  | Hepatitis C virus | 6 | 1 | 1 |
|  | Herpes simples virus | 2 | 1 | 1 |
|  | *Histoplasma capsulatum* | 1 | 0 | 0 |
|  | Human endogenous retroviruses | 4 | 1 | 1 |
|  | Human herpes virus-6 | 2 | 1 | 1 |
|  | Human immunodeficency virus | 7 | 2 | 2 |
|  | Human retrovirus-5 | 3 | 2 | 1 |
|  | Human T-lymphotropic virus | 7 | 2 | 2 |
|  | Intracisternal A-type particles | 2 | 1 | 1 |
|  | *Kingella kingae* | 1 | 0 | 0 |
|  | *Klebsiella pneumoniae* | 1 | 0 | 0 |
|  | Measles virus | 2 | 1 | 2 |
|  | *Mycobacterium spp.* | 3 | 1 | 2 |
|  | *Mycoplasma* spp. | 3 | 0 | 1 |
|  | *Neisseria gonorrhoeae* | 3 | 0 | 1 |
|  | *Nocardia brasiliensis* | 1 | 1 | 1 |
|  | Parainfluenza virus | 1 | 1 | 1 |
|  | Parvovirus B19 | 6 | 2 | 1 |
|  | *Pasteurella multocida* | 1 | 0 | 0 |
|  | *Penicillium marneffei* | 1 | 0 | 1 |
|  | Poliovirus | 2 | 1 | 1 |
|  | *Porphyromonas gingivalis* | 2 | 0 | 1 |
|  | *Proteus mirabilis* | 1 | 2 | 1 |
|  | *Pseudomonas aeruginosa* | 1 | 0 | 0 |
|  | Ross River virus | 1 | 0 | 1 |
|  | Rubella virus | 1 | 0 | 1 |
|  | *Salmonella* spp. | 5 | 1 | 2 |
|  | *Scedosporium apiospermum* | 1 | 1 | 2 |
|  | *Schistosoma spp.* | 1 | 0 | 2 |
|  | *Shigella* spp. | 3 | 0 | 1 |
|  | Sindbis virus | 1 | 0 | 1 |
|  | *Staphylococcus aureus* | 4 | 1 | 2 |
|  | *Streptococcus* spp. | 6 | 1 | 1 |
|  | *Toxoplasma gondii* | 3 | 3 | 3 |
|  | *Ureaplasma urealyticum* | 3 | 0 | 1 |
|  | *Wuchereria bancrofti* | 1 | 0 | 1 |
|  | *Yersinia* spp. | 4 | 2 | 3 |
| Genitourinary system | *Alliococcus otitidis* | 1 | 0 | 1 |
|  | *Candida albicans* | 2 | 1 | 2 |
|  | *Chlamydia trachomatis* | 7 | 3 | 4 |
|  | Epstein-Barr virus | 1 | 1 | 2 |
|  | *Escherichia coli* | 3 | 1 | 2 |
|  | Hepatitis B virus | 3 | 2 | 3 |
|  | Hepatitis C virus | 3 | 1 | 3 |
|  | Human immunodeficiency virus | 2 | 1 | 2 |
|  | Human papillomavirus | 1 | 1 | 1 |
|  | *Mycobacterium tuberculosis* | 1 | 0 | 2 |
|  | *Neisseria gonorrhoeae* | 1 | 0 | 1 |
|  | Parvovirus B19 | 1 | 1 | 1 |
|  | *Pseudomonas aeruginosa* | 1 | 1 | 2 |
|  | *Schistosoma* spp. | 2 | 0 | 2 |
|  | *Treponema pallidum* | 1 | 0 | 1 |
| Pregnancy | *Chlamydia trachomatis* | 2 | 1 | 2 |
|  | *Coxiella burnetii* | 2 | 3 | 2 |
|  | Cytomegalovirus | 1 | 0 | 1 |
| Perinatal blood | Adenovirus | 1 | 0 | 1 |
|  | *Aggregatibacter actinomycetemcomitans* | 1 | 0 | 1 |
|  | *Bacteroides* spp. | 2 | 0 | 1 |
|  | *Borrelia burgdorferi* | 1 | 0 | 1 |
|  | *Campylobacter rectus* | 1 | 0 | 1 |
|  | *Capnocytophaga* spp. | 1 | 0 | 1 |
|  | *Chlamydia trachomatis* | 1 | 0 | 1 |
|  | *Coxiella burnetii* | 2 | 3 | 2 |
|  | *Cytomegalovirus* | 7 | 0 | 2 |
|  | Epstein-Barr virus | 1 | 0 | 0 |
|  | *Escherichia coli* | 2 | 1 | 3 |
|  | *Fusobacterium nucleatum* | 1 | 0 | 1 |
|  | *Gardnerella vaginalis* | 3 | 1 | 3 |
|  | *Haemophilus influenzae* | 1 | 0 | 1 |
|  | *Helicobacter pylori* | 1 | 0 | 1 |
|  | Hepatitis B virus | 1 | 0 | 1 |
|  | Herpes simplex virus | 2 | 0 | 1 |
|  | Human immunodeficiency virus | 2 | 0 | 1 |
|  | Human papillomavirus | 1 | 0 | 1 |
|  | *Listeria monocytogenes* | 1 | 1 | 3 |
|  | *Mobiluncus spp.* | 1 | 0 | 0 |
|  | *Moraxella catarrhalis* | 1 | 0 | 1 |
|  | *Mycoplasma hominis* | 3 | 1 | 3 |
|  | *Neisseria gonorrhoeae* | 1 | 0 | 1 |
|  | *Porphyromonas gingivalis* | 1 | 0 | 1 |
|  | *Prevotella bivis* | 2 | 0 | 1 |
|  | Rubella virus | 2 | 0 | 2 |
|  | Severe acute respiratory syndrome virus | 2 | 0 | 1 |
|  | *Staphylococcus aureus* | 1 | 0 | 3 |
|  | *Streptococcus agalactiae* | 2 | 1 | 3 |
|  | *Toxoplasma gondii* | 3 | 0 | 2 |
|  | *Treponema denticola* | 1 | 0 | 1 |
|  | *Trichomonas vaginalis* | 1 | 0 | 1 |
|  | *Ureaplasma urealyticum* | 3 | 1 | 3 |
|  | Varicella zoster virus | 2 | 0 | 1 |
| Congenital malformations | *Aspergillus ochraceus* | 1 | 0 | 1 |
|  | Fusarium spp. | 1 | 0 | 1 |
|  | Rubella virus | 3 | 1 | 1 |
| Symptoms not classified elsewhere | Adenovirus | 1 | 0 | 1 |
|  | *Aggregatibacter actinomycetemcomitans* | 1 | 1 | 0 |
|  | *Aspergillus spp.* | 5 | 2 | 3 |
|  | *Bordetella spp.* | 5 | 2 | 2 |
|  | *Candida albicans* | 1 | 1 | 0 |
|  | *Chlamydia pneumoniae* | 4 | 1 | 1 |
|  | *Claviceps purpura* | 1 | 0 | 1 |
|  | *Cryptococcus neoformans* | 1 | 1 | 0 |
|  | Cytomegalovirus | 1 | 0 | 1 |
|  | *Echincoccus* spp. | 1 | 0 | 1 |
|  | *Enterobius vermicularis* | 1 | 1 | 2 |
|  | *Erysipelothrix rhusiopathiae* | 1 | 1 | 2 |
|  | *Escherichia coli* | 1 | 2 | 0 |
|  | *Fasciola hepatica* | 1 | 1 | 2 |
|  | *Geotricum candidum* | 1 | 1 | 0 |
|  | *Haemophilus influenzae* | 2 | 1 | 2 |
|  | *Helicobacter pylori* | 1 | 1 | 1 |
|  | Hepatitis B virus | 1 | 0 | 1 |
|  | Hepatitis C virus | 1 | 0 | 1 |
|  | Human herpesvirus-8 | 1 | 0 | 1 |
|  | Human immunodeficency virus | 3 | 2 | 1 |
|  | Klebsiella pneumoniae | 1 | 1 | 0 |
|  | Measles virus | 1 | 0 | 1 |
|  | *Moraxella catarrhalis* | 1 | 1 | 2 |
|  | *Morganella morganii* | 1 | 1 | 0 |
|  | *Mycobacterium tuberculosis* | 3 | 1 | 1 |
|  | *Mycoplasma* spp. | 2 | 2 | 1 |
|  | *Paragonimus* spp. | 1 | 0 | 1 |
|  | *Pneumocystis jiroveci* | 1 | 1 | 0 |
|  | *Proteus mirabilis* | 1 | 1 | 0 |
|  | *Pseudomonas aeruginosa* | 3 | 1 | 2 |
|  | *Scedosporium apiospermum* | 1 | 1 | 2 |
|  | *Staphylococcus aureus* | 2 | 1 | 2 |
|  | *Streptococcus pneumoniae* | 2 | 1 | 2 |
|  | *Strongyloides stercoralis* | 1 | 1 | 1 |
|  | *Taenia solium* | 1 | 0 | 1 |
|  | *Trichoderma harzianum* | 1 | 2 | 3 |
|  | *Ureaplasma urealyticum* | 1 | 0 | 1 |
|  | Varicella zoster virus | 2 | 0 | 1 |
|  | *Wuchereria bancrofti* | 1 | 2 | 0 |

Note: The assessment of the number of Koch’s postulates and Hill’s criteria fulfilled includes the total evidence base in the systematic review; it is not only based on the evidence from level IV studies

*studies reporting no association between the infectious agent and the chronic condition have also been identified
