# Supplementary material for: Causal Inference Regarding Infectious Aetiology of Chronic Conditions: A Systematic Review
Source: PLoS One. 2013 Jul 25;8(7):e68861. doi: 10.1371/journal.pone.0068861 (PMC3723854; doi:10.1371/journal.pone.0068861)
Supplement: Table S4 — Grouping of infectious agents into classes (i.e., bacteria, viruses, fungi, parasites). (DOCX) [file pone.0068861.s004.docx]

**SUPPLEMENT C: GROUPING OF INFECTIOUS AGENTS**

Table 4: Grouping of infectious agents into classes (i.e., bacteria, viruses, fungi, parasites)

| **Species** | **Abbreviation (if applicable)** | **Class** |
| --- | --- | --- |
| *Acanthamoeba polyphaga* |  | Parasite |
| Adenovirus |  | Virus |
| *Aspergillus* spp*.* |  | Fungi |
| *Bacillus cereus* |  | Bacterium |
| Bovine Kidney virus | BK virus | Virus |
| *Blastocystis hominis* |  | Parasite |
| *Blastomyces dermatitidis* |  | Fungus |
| *Bordetella* spp*.* |  | Bacteria |
| *Campylobacter jejuni* |  | Bacterium |
| *Candida* spp*.* |  | Fungi |
| Chikungunya virus |  | Virus |
| *Chlamydia* spp*.* |  | Bacteria |
| Cytomegalovirus | CMV | Virus |
| *Demodex folliculorum* |  | Parasite |
| Dengue virus |  | Virus |
| *Eikenella corrodens* |  | Bacterium |
| *Entamoeba coli* |  | Parasite |
| *Enterobacter* spp*.* |  | Bacterium |
| *Enterococcus faecalis* |  | Bacterium |
| Enterovirus |  | Virus |
| Epstein-Barr Virus | EBV | Virus |
| *Escherichia coli* |  | Bacterium |
| Filarial nematodes |  | Parasites |
| *Geotrichum candidum* |  | Fungus |
| *Giardia* spp*.* |  | Parasites |
| *Haemophilus influenzae* |  | Bacterium |
| *Helicobacter* *pylori* |  | Bacterium |
| Hepatitis A-G Virus | HAV, HBV, HCV, etc. | Virus |
| Herpes Simplex Virus | HSV | Virus |
| *Histoplasma capsulatum* |  | Fungus |
| Human Herpes Virus | HHV | Virus |
| Human Immunodeficiency Virus | HIV | Virus |
| Human Papillomavirus | HPV | Virus |
| Influenza virus |  | Virus |
| *Klebsiella* spp. |  | Bacteria |
| Measles virus |  | Virus |
| *Moraxella catarrhalis* |  | Bacterium |
| *Mycobacterium* spp*.* |  | Bacteria |
| *Mycoplasma* spp. |  | Bacteria |
| *Neisseria gonorrhoeae* |  | Bacteria |
| *Orientia tsutsugamushi* |  | Bacterium |
| Parainfluenza virus |  | Virus |
| *Penicillium* spp*.* |  | Fungi |
| Poliovirus |  | Virus |
| *Proprionibacterium acnes* |  | Bacterium |
| *Proteus mirabilis* |  | Bacterium |
| *Pseudomonas aeruginosa* |  | Bacterium |
| Respiratory Syncytial Virus | RSV | Virus |
| Rhinovirus |  | Virus |
| *Rhizopus nigricans* |  | Fungus |
| *Rickettsia spp.* |  | Bacteria |
| Rotavirus |  | Virus |
| Rubella virus |  | Virus |
| *Salmonella spp.* |  | Bacteria |
| *Schistosoma* spp. |  | Parasites |
| *Serratia marcescens* |  | Bacterium |
| *Staphylococcus* spp. |  | Bacterium |
| *Streptococcus* spp. |  | Bacterium |
| *Toxocara* spp*.* |  | Parasites |
| *Toxoplasma gondii* |  | Parasite |
| Transfusion Transmitted Virus | TTV | Virus |
| *Treponema spp.* |  | Bacteria |
| *Trichomonas vaginalis* |  | Parasite |
| *Tropheryma whipplei* |  | Bacterium |
| *Trypanosoma cruzi* |  | Parasite |
| *Ureaplasma urealyticum* |  | Bacterium |
| Vaccinia virus |  | Virus |
| Varicella Zoster Virus | VZV | Virus |
| *Vibrio cholerae* |  | Bacterium |
| *Wuchereria bancrofti* |  | Parasite |

Note: Viruses act intracellularly and are able to utilise the information system of the host cells and act on the DNA, RNA, or protein level. Interpretation of the results in Figure 8 suggests that viruses are the most versatile agents as they were capable of causing disease in every organ system included in this review. Also, when analysing the chronic conditions most frequently identified in the current review, viruses were most often implicated in the aetiology, followed by bacteria, fungi, and parasites. There were several different bacterial genera identified in this review, and they were capable of causing disease in almost every disease area. Bacteria may generally be associated with organs that are exposed to the “exterior”, such as the respiratory tract. They may inflict damage by secreting enzymes or toxins that interfere with biochemical processes of cells or organ systems (e.g., shiga or cholera toxins and superantigens). There were a limited number of parasites identified in the review, but they were capable of causing disease in many organ systems. Parasites are highly specialised and adapted to their individual host and they can have a number of adverse impacts by replicating in certain organs and tissues, by secreting toxic substances or merely due to their size. There were also a small number of fungal genera identified in this dataset. Fungi are also often associated with organs exposed to the “exterior” surfaces of the body and, in the current data-set; they seemed to have a propensity to be associated with the respiratory tract.
